# Longitudinal dynamics and site-specific recovery of the human respiratory microbiome following smoking cessation

**DOI:** 10.1186/s12931-026-03644-z

**Published:** 2026-04-02

**Authors:** Silvia Gschwendtner, Draginja Kovacevic, Karoline I. Gaede, Christian Herzmann, Jörg Overmann, Michael Schloter, Susanne Krauss-Etschmann

**Affiliations:** 1 Research Unit of Comparative Microbiome Analysis, Helmholtz Munich, German Research Center for Environmental Health, Neuherberg, Germany; 2 Early Life Origins of Chronic Lung Disease, Research Center Borstel - Leibniz Lung Center, Airway Research Center North (ARCN), German Center for Lung Research (DZL), Borstel, Germany; 3 DZL-Laboratory for Experimental Microbiome Research, Research Center Borstel, Airway Research Center North (ARCN), German Center for Lung Research (DZL), Borstel, Germany; 4 BioMaterialBank Nord, Research Center Borstel - Leibniz Lung Center, Borstel, Airway Research Center North (ARCN), German Center for Lung Research (DZL), Borstel, Germany; 5 Public Health Authority Stormarn, Environmental Health Department, Bad Oldesloe, Germany; 6 Clinical Trials Unit, German Center for Infection Research (DZIF), Borstel, Germany; 7 Bavarian State Collections of Natural History, Munich, Germany; 8 Chair for Molecular Diversity Research, Faculty of Biology, Ludwig-Maximilians-Universität München, Munich, Germany; 9 Chair for Environmental Microbiology, Technical University of Munich, Munich, Germany; 10 German Center for Lung Research (DZL), Comprehensive Pneumology Center (CPC-M), Munich, Germany; 11 Institute for Experimental Medicine, Christian-Albrechts-Universität zu Kiel, Kiel, Germany; 12 PopGen 2.0 Biobanking Network (P2N), Kiel University, University Hospital Schleswig-Holstein, Campus Kiel, Kiel, Germany

**Keywords:** Smoking, Microbiome, Smoking cessation, Respiratory system, Dysbiosis

## Abstract

**Background:**

The human respiratory tract harbours diverse microbial communities crucial for health, but their dynamics during environmental perturbations like smoking remain poorly understood. While smoking is a major risk factor for various diseases, its compartment-specific effects on the respiratory microbiome and potential recovery following cessation have not been fully elucidated. Here, we present a longitudinal, multi-site study of respiratory microbiome dynamics in smokers undergoing cessation, benchmarked against healthy never-smokers.

**Methods:**

Using standardized sampling of the anterior nares, oropharynx, and bronchoalveolar lavage (BAL), combined with 16S rRNA gene amplicon sequencing and rigorous contamination controls, we characterized community composition, diversity, inter-individual variability, and microbial interactions across airway compartments.

**Results:**

Smokers exhibited pronounced microbiome alterations: nasal richness increased, while lung richness and core taxa were decreased. Smoking-induced changes were compartment-specific and most pronounced in nose and lung. The degree of individual-specific differences in community structure was elevated in smokers and correlated with smoking history. Short-term cessation (6 weeks) led to minor shifts in genus abundance but increased similarity between oropharyngeal and lung communities, whereas long-term cessation (1 year) resulted in partial restoration, particularly in lung and nasal microbiomes. Some genera, including *Haemophilus* and *Prevotella_7*, showed persistent alterations, suggesting lasting smoking effects. Network analyses revealed that smoking disrupted microbial co-occurrence and reduced community connectivity, whereas cessation partially restored interaction networks, with central taxa remaining altered and dynamics differing between oropharynx and lung, reflecting different underlying ecological assembly processes. Recovery trajectories were highly individualized, with lung microbiomes influenced by deterministic processes and upper airway microbiomes shaped by stochastic factors, explaining site-specific responses and the persistence of personalized microbial signatures.

**Conclusion:**

These results provide the first time-resolved, multi-compartment characterization of respiratory microbiome recovery after smoking cessation, revealing that smoking leaves lasting, site-specific imprints on airway microbial communities and interactions. Our findings underscore the need for individual and compartment-specific approaches when designing microbiome-based interventions to support respiratory health.

**Supplementary Information:**

The online version contains supplementary material available at 10.1186/s12931-026-03644-z.

## Background

Human-associated microbial communities have co-evolved with their host over millions of years, colonizing nearly all body surfaces, including the respiratory tract (RT). The RT comprises the upper respiratory tract (URT; nose, sinuses, naso- and oropharynx, supraglottic larynx) and the lower respiratory tract (LRT; infraglottic larynx, trachea, lungs). In adults, the airways cover ~ 70 m² [1] and are populated by compartment-specific microbiota essential for RT function and host health. Community composition of the RT reflects a dynamic balance between immigration (from the oral cavity and URT), elimination (via mucociliary clearance, cough, and immunity), and differential growth [2, 3].

The anterior nares, lined by keratinized epithelium, harbour typical skin colonizers (*Corynebacterium*, *Propionibacterium*, *Staphylococcus*) alongside respiratory-associated genera like *Dolosigranulum*, *Moraxella*, and *Streptococcus* [4]. These organisms contribute to early-life colonization and help protecting the host from pathogenic invasion: *Dolosigranulum* and *Corynebacterium* are linked to reduced *Streptococcus pneumoniae* colonization [5, 6], and *Staphylococcus epidermidis* can inhibit *S. aureus* biofilm formation [7]. The nasopharynx has overlapping but more diverse genera, including *Haemophilus*, while the oropharynx is enriched in *Streptococcus*, *Neisseria*, *Rothia*, and anaerobes such as *Leptotrichia*, *Prevotella* and *Veillonella*. Together, these genera help to maintain a healthy microbial balance by contributing to mucosal immunity and interacting with the host’s immune system [8, 9].

Although long considered sterile, the healthy LRT has been recently recognized to harbour a low biomass yet diverse microbial community, dominated by *Prevotella*, *Streptococcus*, *Veillonella*, *Actinomyces*, *Fusobacterium*, and *Haemophilus* [10–14]. These microbes modulate immune responses and maintain pulmonary homeostasis [10, 11].

Smoking is a major risk factor for lung cancer, chronic obstructive pulmonary disease (COPD), cardiovascular disease, and periodontitis [15]. Increasing evidence suggests that smoking-induced dysbiosis of the respiratory microbiome may contribute to disease initiation and progression [16] possibly via altered oxygen tension, immune suppression, enhanced biofilm formation, and exposure to smoke-derived chemicals or bacteria present in cigarettes [16–19].

However, reports how smoking affects respiratory microbiota remain inconsistent and site dependent. Some studies observed increased diversity in the oral cavity and URT [13, 20–22], while others reported decreased diversity [23, 24]. In contrast, reduced diversity is more consistently observed in the lower airways [13, 25]. Although smoking-induced shifts in microbial composition are commonly detected, the specific taxa involved vary considerably, limiting their predictive value.

Notably, some smoking-associated alterations appear reversible. In mice, oropharyngeal microbial diversity and composition normalized within three months of smoking cessation [26]. Comparable recovery was observed in the human nasopharynx after 12–15 months [27]. However, persistent changes in the LRT [25] suggest compartment-specific and potentially long-lasting effects on the airway microbiome, even in healthy individuals.

Although evidence suggests that partial restoration of a healthy microbiome may occur after smoking cessation, the temporal dynamics across distinct RT compartments and the extent of inter-individual variability remain poorly understood.

Here, we present longitudinal microbiome profiles from multiple respiratory sites during smoking cessation, benchmarked against healthy never-smokers. These data enable time-resolved analysis of microbiome recovery across the respiratory tract and uncover personalized recovery trajectories. Our findings establish a basis for targeted microbiome-based interventions, particularly during early phases of smoking cessation, when microbial communities are most perturbed and only partially restored.

## Materials and methods

### Participant selection and sampling

The LuMEn cohort was recruited at the Center for Clinical Studies, Research Center Borstel, Germany, between March 2017 and July 2020 as described previously [14]. Eligible participants were Caucasian adults (≥ 18 years) who met the following criteria: absence of (1) respiratory infection or systemic glucocorticoid therapy within the past month, (2) antibiotic therapy within the past two months, (3) diabetes mellitus, (4) pregnancy or lactation, (5) active or previous tuberculosis, (6) immunosuppression, or (7) known pulmonary disease, except for COPD stage GOLD I/II; however, no participant with COPD was ultimately enrolled. Individuals with medical contraindications to bronchoscopy, as determined by the investigator (e.g., allergy to sedative agents), were excluded. Data were recorded using a structured, study-specific questionnaire including items on sociodemographic characteristics, smoking history (including passive smoking exposure and use of electronic cigarettes or vaping devices) and cessation behavior, medical history, pet contact, alcohol consumption, and environmental exposures. Demographic characteristics are presented in Table 1. Based on CDC definitions, participants were categorized as active smokers (AS), former smokers (FS), or never-smokers (NS).

**Table 1:** Summary of baseline characteristics of the study cohort, including never-smokers (NS), active smokers (AS) and former smokers 6 weeks post-cessation (FS6w), and 1-year post-cessation (FS1y)

Characteristic	Total	NS	AS	FS6w	FS1y	
	*n* = 25 ^1^	*n* = 10 ^1^	*n* = 15 ^1^	*n* = 15 ^1^	*n* = 5 ^1^	*p* value ^2^
Age	39 (13)	37 (14)	40 (13)	40 (13)	37 (15)	0.454
Female	11 (44%)	6 (60%)	5 (33%)	5 (33%)	2 (40%)	0.241
Height	176 (11)	172 (10)	178 (12)	178 (12)	172 (12)	0.266
Weight	81 (16)	82 (11)	81 (20)	84 (18)	84 (28)	0.374
Nicotine	269 (475)	2 (1)	447 (550)	3 (2)	2 (0)	< 0.001
Cotinine	634 (713)	5 (10)	1,053 (633)	8 (14)	2 (0)	< 0.001
Anabasine	5 (4)	2 (0)	7 (5)	2 (0)	2 (0)	< 0.001
OH_Cotinine	3,088 (4,788)	108 (215)	5,075 (5,357)	65 (52)	40 (0)	< 0.001
Smoking years	14 (15)	0 (0)	24 (13)	24 (13)	21 (15)	n.a.
CPD	9 (8)	0 (0)	15 (5)	15 (5)	16 (6)	n.a.
CPD max	18 (16)	0 (0)	29 (9)	29 (9)	29 (9)	n.a.
Pack years	13 (18)	0 (0)	21 (19)	21 (19)	21 (27)	n.a.
Allergy	10 (40%)	4 (40%)	6 (40%)	2 (13%)	0 (0%)	0.999
Hayfever	6 (24%)	2 (20%)	4 (27%)	0 (0%)	0 (0%)	0.999
Pet contact	17 (68%)	7 (70%)	10 (67%)	10 (67%)	3 (60%)	0.999
Alcohol	13 (52%)	6 (60%)	7 (47%)	7 (47%)	3 (60%)	0.688
Pollutants	4 (16%)	1 (10%)	3 (20%)	1 (6.7%)	1 (20%)	0.627

^2^ *p* values calculated for independent groups (NS and AS) via Kruskal–Wallis test and Fisher’s exact test, respectively

^1^ Mean (SD); n (%)

The sub-cohort for the smoking cessation analysis was not preselected but rather depended on participants’ willingness to engage in the smoking cessation program and their success in completing it. Participants who successfully completed the cessation program at any of the designated timepoints were included in this sub-cohort. Due to the non-random nature of this selection, a formal power calculation was not performed prior to the study.

Nicotine, cotinine, anabasine, and OH-cotinine concentrations were quantified in unprocessed urine samples using liquid chromatography-tandem mass spectrometry (LC-MS/MS). Quality controlled biosampling included deep bilateral nasal swabs (*n* = 36), bilateral oropharyngeal swabs (*n* = 45), and bronchoalveolar lavage (BAL; *n* = 45) representing both important URT and LRT compartments. Nasal and oropharyngeal swabs (Mast swab TM, Mast Group Ltd., UK) were immediately frozen in cryotubes on dry ice. Flexible bronchoscopy was performed in accordance with German guidelines. The bronchoscope was advanced into a subsegmental bronchus of the middle lobe, and BAL was carried out with 15 aliquots of 20 mL sterile saline (0.9%), for a total of 300 mL. The first BAL fraction was discarded due to possible contamination, while the remaining fractions were pooled and stored at − 80 °C. Sterile saline solution was included as a control for microbiome analyses.

The study was approved by the Ethics Committee of the University of Luebeck (ref. 16–145) and registered at clinicaltrials.gov (NCT03562442; first submitted: May 26, 2018). Oral and written informed consent was obtained from all participants in accordance with ICH/GCP guidelines.

### Microbial community analysis

Details on microbiome sample processing and subsequent data analysis are provided in Additional Material 1. Briefly, DNA extraction was performed using the PureLink™ Genomic DNA Mini Kit (ThermoFisher Scientific, Altham, USA) according to the manufacturer’s protocol for Gram-positive bacteria. To identify contaminants deriving from sampling and extraction kit, controls were included (DNA extraction of 5 mL sterile saline solution (8 samples) and 5 mL bronchoscope flushing (8 samples), as well as 5 blank extractions).

Amplicon sequencing of the V4 hypervariable region of the 16S rRNA gene was performed on a MiSeq Illumina instrument (MiSeq Reagent Kit v3 (600 Cycle); Illumina, San Diego, CA, USA) using the universal eubacterial primers 515F [28] and 806R [29]. The sequence data obtained in this study are deposited in the short-read archive of NCBI under accession number PRJNA1328433.

Sequence processing was performed using DADA2 v 1.30 [30], followed by taxonomic assignment via SILVA v138.1. Reads were excluded if classified as mitochondria or chloroplast or if the phylum was missing. All blank samples (saline solution, bronchoscope flushing, blank extraction and PCR no template control) were analysed together with biological samples and used to identify and remove potential contaminants from the dataset (in total: 45 sequences; see Additional Table 1). After decontamination, the dataset comprised 5,446,191 reads (corresponding to an average of 43,223 reads per sample) assigned to 2,221 amplicon sequence variants (ASV).

All plots and statistics were performed in R version 4.4.0 (https://www.R-project.org). Sequencing data were normalized using cumulative-sum scaling (CSS) via the metagenomeSeq R package [31]. The subsequent bioinformatic analysis comprised assessment of alpha diversity, overall community composition (beta diversity including recovery following smoking cessation), core microbiome composition, inter-individual variability, differential abundance analysis (DESeq2), associations between inter-individual variability, smoking-related factors and microbial genera, microbial community assembly processes (ßNTI and RCbray framework), and bacterial co-occurrence networks (NetCoMi). Detailed methodological descriptions are provided in Additional Material 1.

## Results

### Cohort characteristics

The study comprised 25 participants (10 never smokers (NS) and 15 active smokers (AS)). Samples after cessation were taken at 6 weeks (FS6w; 15 subjects) and 1 year (FS1y; 5 subjects because only those completed 1 year cessation). The baseline data are summarized in Table 1. Demographic characteristics (age, sex, height, and weight) and clinical and environmental data (allergy status, hay fever, pet contact, alcohol consumption, and pollutant exposure) did not differ between NS and AS (all *p* > 0.05). Biomarkers of nicotine exposure (nicotine, cotinine, anabasine, and OH-cotinine) were significantly higher in AS compared with NS (all *p* < 0.001), while former smokers show low levels. Two AS participants reported passive smoking exposure; due to its very low frequency and absence of corresponding elevations in cotinine levels, passive smoking was not considered in the subsequent analyses. The use of electronic cigarettes or vaping devices was not indicated by any participant.

### Respiratory tract microbial composition in never-smokers (NS) and active smokers (AS)

**Fig. 1:**
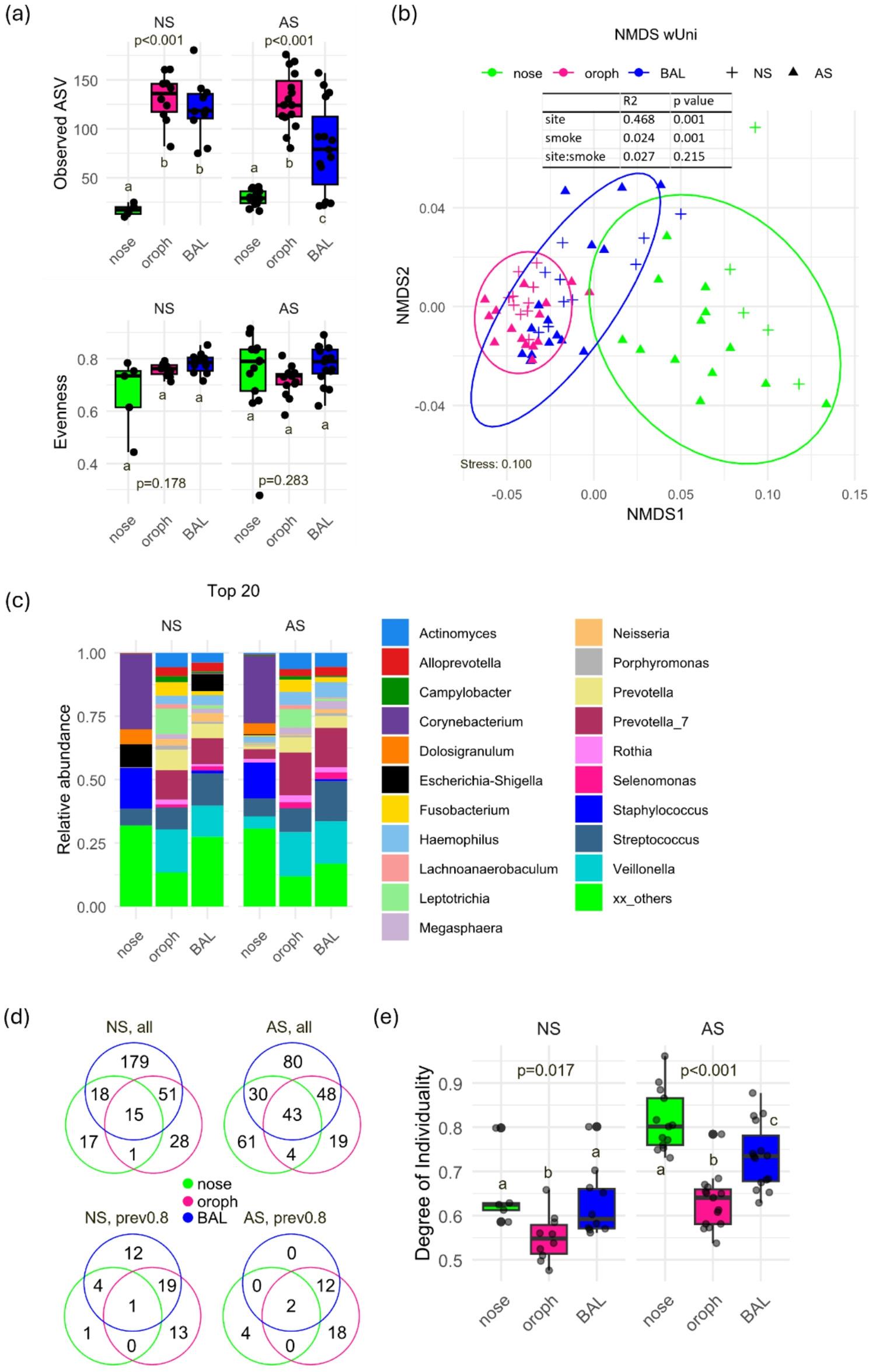
*Microbial communities among the respiratory tract of never-smokers (NS) and active smokers (AS)* **a** Boxplots of alpha diversity measures showed decreasing richness oropharynx>BAL>nose in both never- (NS, *n*=10 (nose: *n*=5)) and active smokers (AS, *n*=15), while evenness did not differ. Different letters (a, b, c) indicate statistically significant differences between groups; groups that do not share a letter differ significantly (*p* < 0.05). Significance was calculated via generalized linear mixed-effect models including the study subjects as random effect term with Benjamini-Hochberg p adjustment. Pairwise comparisons were performed using estimated marginal means (emmeans). **b** NMDS plot of weighted UniFrac distances revealed distinct microbial communities in all airway cavities and a significant effect of smoking, calculated via PERMANOVA using strata to restrict permutations within subjects with Benjamini-Hochberg correction. **c** Relative abundance of the top 20 genera showed overlap in abundant genera between oropharynx and BAL, while nose clearly differed, with smoking-related changes across respiratory sites. **d** Venn diagrams showed number of shared and unique genera for nose, oropharynx and BAL samples in never- (NS, *n*=10 (nose: *n*=5)) and active smokers (AS, *n*=15) (top: without prevalence threshold, bottom: prevalence 0.8). **e** Degree of individuality increased with smoking and was lowest in oropharynx in both never- (NS, *n*=10 (nose: *n*=5)) and active smokers (AS, *n*=15). Different letters (a, b, c) indicate statistically significant differences between groups; groups that do not share a letter differ significantly (*p* < 0.05). Significance was calculated via generalized linear mixed-effect models including the study subjects as random effect with Benjamini-Hochberg correction for multiple comparisons. Pairwise comparisons were performed using estimated marginal means (emmeans). Detailed model results are shown in Additional Table 4.

Across both NS and AS, microbial diversity varied significantly across respiratory compartments, with observed ASV decreasing from oropharynx to lung to nose, while evenness remained similar (Fig. 1a). Smoking altered this gradient site-specific, with observed ASV being reduced in BAL (*p* = 0.041), increased in the nose (*p* = 0.009), and unchanged in the oropharynx (Fig. 1a). Beta diversity confirmed distinct, compartment-specific communities significantly affected by smoking (Fig. 1b).

For NS, the nose was dominated by *Corynebacterium*,* Dolosigranulum*,* Escherichia-Shigella*,* Streptococcus*, and *Staphylococcus*. For AS, the nasal community shifted toward higher abundances of *Haemophilus*,* Mycoplasma*,* Prevotella* spp., and *Veillonella*, with reduced *Escherichia-Shigella* (Fig. 1c, Additional Fig. 1). Smoking was associated with a higher number of total genera in this compartment (NS: 51 vs. AS: 138, Fig. 1d). Nasal inter-individual variability was high in both groups but further increased in AS (*p* < 0.001) (Fig. 1e).

For both NS and AS, the oropharynx was characterized by *Actinomyces*,* Fusobacterium*,* Leptotrichia*,* Prevotella* spp., *Streptococcus*, and *Veillonella*. Although the number of observed ASV remained stable with smoking, relative genus abundances shifted, including alterations in *Neisseria*,* Prevotella_7* (a distinct subgroup within the *Prevotella* genus, defined by sequence similarity but lacking further taxonomic classification), *Selenomonas*,* Streptococcus*, and *Veillonella* (Fig. 1c, Additional Fig. 1). Compared to other sites, inter-individual variability was lowest in the oropharynx but increased with smoking (*p* = 0.019) (Fig. 1e) and was positively correlated with smoking history (Additional Fig. 1).

BAL samples shared a core microbiome with the oropharynx, dominated by *Actinomyces*,* Prevotella spp.*,* Streptococcus*, and *Veillonella*, but enrichment of *Escherichia-Shigella* in NS. Smoking was accompanied by losses of ASV related to the *Escherichia-Shigella* cluster, increased levels of *Prevorella_7* and *Streptococcus*, shifts in low-abundance genera (e.g. reduced *Alistipes*, Muribaculaceae, and Prevotellaceae UCG-001; increased *Dialister*,* Filifactor*, and *Treponema*) (Fig. 1c, Additional Fig. 1), and markedly reduced genera observed (NS: 263 vs. AS: 201) (Fig. 1d). Like for the other respiratory compartments, inter-individual variability further increased in BAL AS (*p* = 0.001) (Fig. 1e) and was positively correlated with smoking- related factors (Additional Fig. 1).

Core microbiome analysis (≥ 80% prevalence) demonstrated limited overlap across compartments in both groups: for NS, only *Streptococcus* was shared across all sites, whereas *Streptococcus* and *Veillonella* were universally present in AS (Fig. 1d, Additional Table 2). Smoking reduced the number of core genera overall and particularly affected BAL uniqueness, with no BAL-specific genera remaining.

In summary, smoking reshaped the microbiota in a site-specific manner, increasing nasal richness, reducing lung richness, diminishing core genera, and enhancing inter-individual variability in relation to smoking exposure. The lung and nose appeared most susceptible to smoking-induced microbial disruption, whereas the oropharynx remained comparatively stable.

### Response to short-term smoking cessation is mediated by respiratory tract cavity

**Fig. 2:**
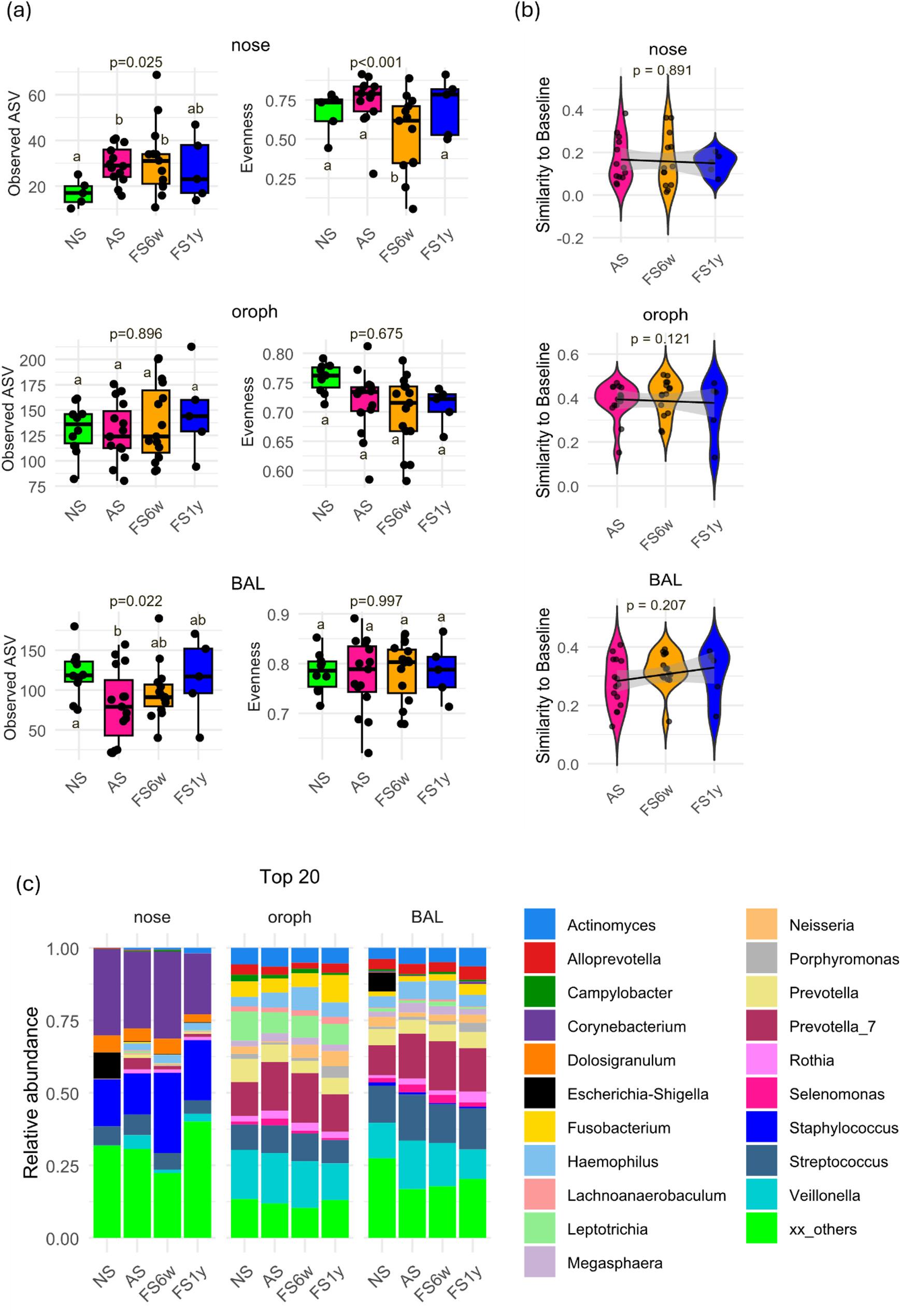
*Smoking and cessation effects on microbiome diversity and composition* **a** Boxplots of alpha diversity measures among never- (NS, *n*=10 (nose: *n*=5)), active (AS, *n*=15) and former smokers (FS6w, *n*=15; FS1y, *n*=5) revealed a highly cavity-dependent response to smoking and cessation, with most pronounced effects on nose (lasting increase of diversity during smoking) and BAL (reduction of diversity during smoking but recovery after cessation). Different letters (a, b, c) indicate statistically significant differences between groups; groups that do not share a letter differ significantly (*p* < 0.05). Significance was calculated via generalized linear mixed-effect models including the study subjects as random effect term, and Benjamini-Hochberg correction for *P* value adjustment. Pairwise comparisons were performed using estimated marginal means (emmeans). **b** Recovery trajectory measured as similarity of the microbial community of active and former smokers towards the NS community (baseline) revealed no significant recovery of community composition after cessation. **c** Relative abundance of the top 20 genera showed site-specific changes for never- (NS, *n*=10 (nose: *n*=5)), active (AS, *n*=15) and former smokers (FS6w, *n*=15; FS1y, *n*=5) in nose, oropharynx and BAL. Detailed model results are shown in Additional Table 4.

After 6 weeks of smoking cessation (FS6w), the richness of the nasal microbiome remained elevated compared to NS, whereas BAL richness partially recovered, and oropharyngeal richness was unchanged (Fig. 2a). Recovery trajectories based on Bray–Curtis distances showed no significant community-level recovery (Fig. 2b). Recovery potential in oropharynx and BAL correlated negatively with smoking duration, pack-years, and cigarette load, while in nose only maximum cigarettes/day was associated (Additional Fig. 3).

At the genus level, only modest shifts were observed: in the nose, *Prevotella* decreased towards NS levels, whereas *Haemophilus*, *Mycoplasma*, *Prevotella_7*, and *Veillonella* remained elevated (Fig. 2c, Additional Fig. 2). In the oropharynx, *Neisseria* and *Selemonas* normalized, while *Prevotella_7*,* Streptococcus* and *Veillonella* remained altered and *Haemophilus* increased. In BAL, *Dialister* and *Neisseria* decreased towards NS level, whereas several low-abundance genera (*Alistipes*, Muribaculaceae, Prevotellaceae UCG-001) remained depleted.

**Fig. 3:**
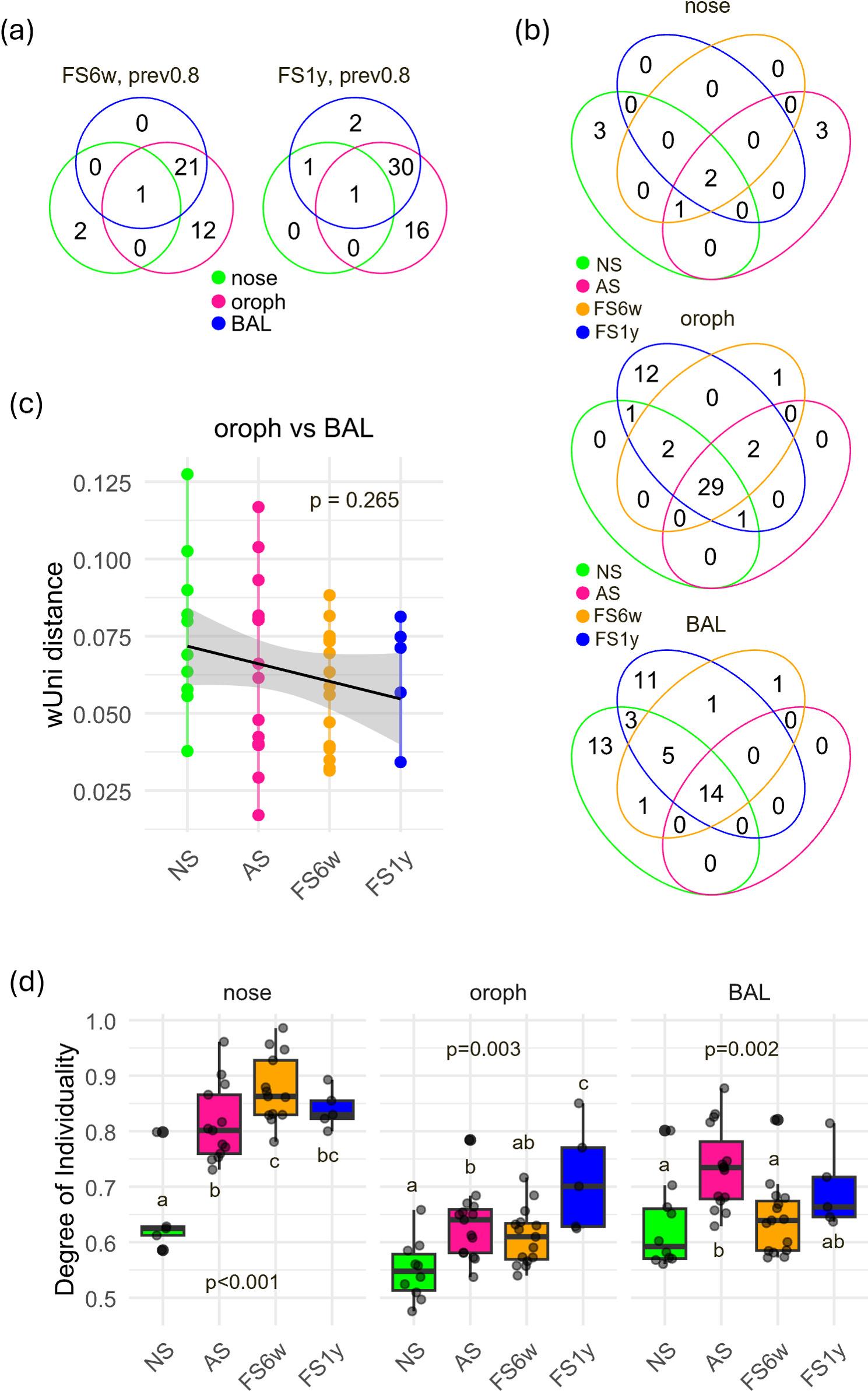
*Smoking and cessation effects on core microbiota and inter-individual variability* **a** Venn diagrams show the number of shared and unique core genera (defined as genera present in at least 80% of subjects per group) between nose, oropharynx and BAL samples after short- (FS6w, *n*=15) and long-term cessation (FS1y, *n*=5). **b** Venn diagrams show number of shared and unique core genera (defined as genera present in at least 80% of subjects per group) between never- (NS, *n*=10 (nose: *n*=5)), active (AS, *n*=15) and former smokers (FS6w, *n*=15; FS1y, *n*=5) for nose, oropharynx and BAL. **c** Comparison of oropharyngeal and BAL communities within subjects (based on weighted Unifrac distances) showed a higher similarity after cessation compared to never-smokers. **d** Degree of individuality revealed a lasting increase of subject-specific variability during smoking, with site-dependent intensity and most pronounced in nose. Different letters (a-c) indicate significant differences (*p*<0.05) between never- (NS, *n*=10 (nose: *n*=5)), active (AS, *n*=15) and former smokers (FS6w, *n*=15; FS1y, *n*=5); groups that do not share a letter differ significantly (*p* < 0.05). Significance was calculated via generalized linear mixed-effect models including the study subjects as random effect term, and Benjamini-Hochberg correction for *P* value adjustment. Pairwise comparisons were performed using estimated marginal means (emmeans). Detailed model results are shown in Additional Table 4.

Core microbiota analysis identified 3, 34, and 22 genera for the nose, oropharynx, and BAL, respectively (Fig. 3a, Additional Table 2). Shared genera between FS6w and NS but absent in AS may represent early markers of short-term microbial recovery following smoking cessation (Fig. 3b, Additional Table 2). Additionally, the oropharyngeal and BAL microbiomes of FS6w showed increased similarity compared to NS (more overlapping core genera (Fig. 3b), decreased UniFrac distances oropharynx-BAL (-0.018, *p* = 0.069) (Fig. 3c, Additional Table 4), and more positive correlations between genera (Additional Fig. 4). Following cessation, the degree of individuality increased in the nose, remained stable in the oropharynx, and normalized in BAL, with the highest levels in the nose (Fig. 3d). While recovery was generally impaired for heavy smokers, inter-individual variability of oropharynx and BAL remained positively associated with smoking history (Additional Fig. 3).

In conclusion, short-term smoking cessation resulted in minor, site-specific recovery of the microbiome, dependent on smoking history, but a notable increase in similarity between the oropharyngeal and lung microbiomes.

### Long-term smoking cessation only leads to a partial and site-specific recovery

After 1 year of cessation (FS1y), nasal richness remained elevated compared to NS, whereas BAL richness normalized towards NS levels (Fig. 2a). No significant community recovery was observed (Fig. 2b), with recovery potential being negatively correlated with smoking history (Additional Fig. 3).

At the genus level, *Veillonella* decreased in the nose towards NS levels, while *Haemophilus*,* Mycoplasma* and *Prevotella_7* remained elevated. In oropharynx, *Haemophilus*,* Prevotella_7*,* Streptococcus* and *Veillonella* normalized, while *Prevotella* decreased compared to NS. In BAL, *Prevotella_7*,* Filifactor* and *Streptococcus* decreased towards NS levels, *Treponema* was still elevated, while *Alistipes*, Muribaculaceae, and Prevotellaceae UCG-001 remained depleted (Fig. 2c, Additional Fig. 2).

Core microbiota analysis identified 2, 47, and 34 genera for nose, oropharynx, and BAL, respectively (Fig. 3a, Additional Table 2), with oropharynx showing 12 unique genera and BAL showing 11, along with greater overlap with NS compared to AS. Across the different respiratory compartments, only *Streptococcus* was shared universally, while 16 genera were oropharynx-specific and 2 were unique for BAL (Fig. 3b). Oropharynx–BAL overlap remained high, supported by continuously reduced between-group distances (-0.022, *p* = 0.087) (Fig. 3c, Additional Table 4).

Variability between individuals remained highest in nose (Fig. 3d). Compared to AS, subject-level variation increased in oropharynx, was stable in nose and BAL, but exceeded NS at all sites, and remained positively correlated with smoking history (Additional Fig. 3).

In conclusion, smoking cessation for 1 year led to partial microbiome recovery, with ongoing high convergence between oropharynx and BAL. High individual variability remained, strongly correlated with smoking history, highlighting the persistent effects of smoking on respiratory microbiota. However, results should be interpreted with caution given the small sample size (*n* = 5).

### Microbial response patterns are highly personalized

**Fig. 4:**
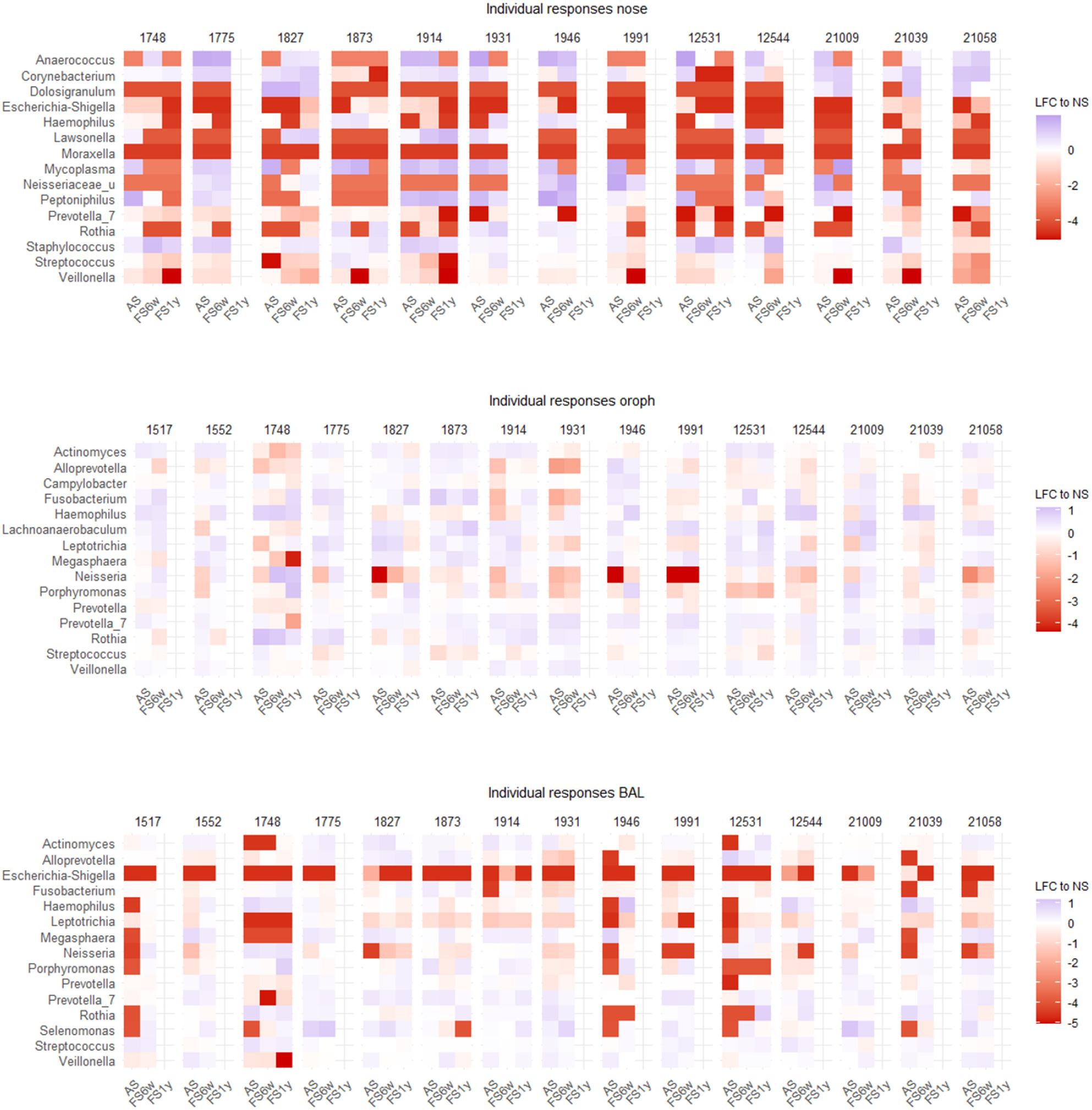
*Individual responses to smoking and cessation* Heatmaps show individual responses of top 15 genera for active (AS) and former smokers (FS6w, FS1y), calculated as log-fold changes to never-smokers baseline.

Beyond general smoking and cessation effects, high inter-individual variability was observed across all airway compartments (Fig. 4). In the nose, some subjects (e.g., 1991, 21058) exhibited persistently high individual variability, with both shared (e.g. *Escherichia-Shigella*) and subject-specific (e.g., *Dolosigranulum*, *Veillonella*) responses to smoking and cessation.

In the oropharynx, personalization was generally lower, although some subjects (1748, 1931) exhibited strong individuality. While smoking induced consistent directional changes in several genera (e.g. *Actinomyces*,* Alloprevotella*,* Prevotella*), responses of others (e.g. *Haemophilus*,* Neisseria*, *Prevotella_7*, *Rothia*, *Veillonella*) varied between subjects and changed differently after cessation.

In BAL, highest inter-individual variability was observed in subjects 1748 and 1946. Despite general trends (e.g., *Alloprevotella*, *Escherichia-Shigella*,* Prevotella*,), multiple genera (e.g. *Actinomyces*,* Haemophilus*, *Neisseria*, *Rothia*, *Streptococcus*) showed subject-specific dynamics partly maintained after cessation.

Concordant patterns between oropharynx and BAL in subjects with high inter-individual variability, such as sustained increases in *Haemophilus* and *Rothia*, further support microbial overlap between these sites and suggest microbial exchange along the respiratory tract.

In summary, smoking and cessation induced both shared and subject-specific microbiome changes across airway compartments, varying by site but with partly similar patterns in oropharynx and BAL.

### Microbial community assembly processes and network topologies

**Fig. 5:**
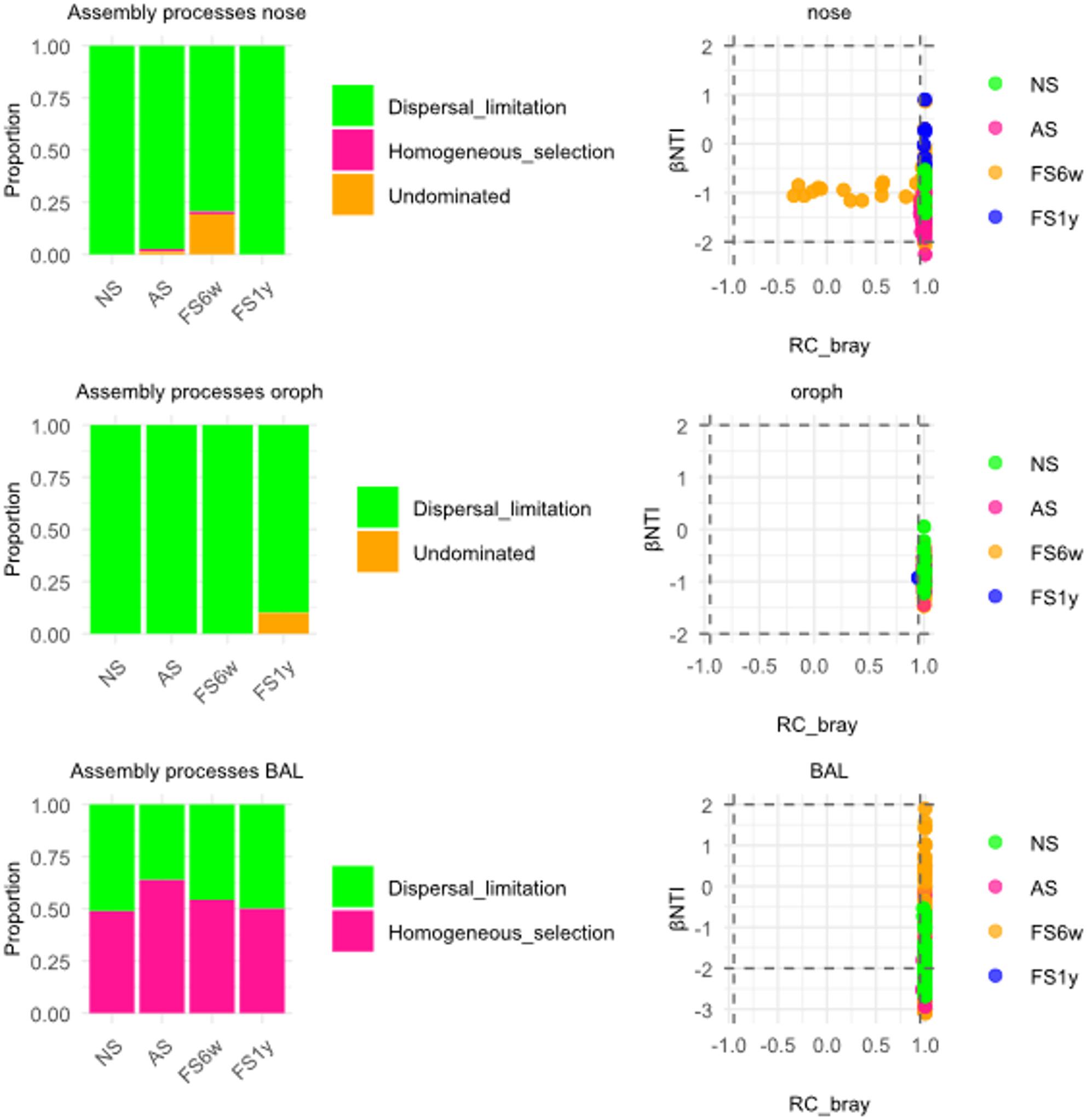
*Community assembly processes in nose, oropharynx and BAL *Differences in ßNTI and RCbray served to determine the processes shaping microbial communities. Analysis showed a dominance of stochastic processes in never- (NS, *n*=10 (nose: *n*=5)), active (AS, *n*=15) and former smokers (FS6w, *n*=15; FS1y, *n*=5) in nose and oropharynx, whereas the BAL community was shaped by both stochastic and deterministic assembly, with temporarily higher contribution of selection in AS and after short-term cessation (FS6w). The thresholds for |βNTI| = 2 and |RCbray| = 0.95 are highlighted as horizontal and vertical dashed lines, respectively.

In the βNTI/RCBray framework, community assembly processes are classified into deterministic and stochastic components, with deterministic processes indicating selection-driven assembly, whereas stochastic processes reflect random dispersal and drift (for details see Additional Material 1). URT microbial community assembly was predominantly shaped by stochastic processes (Fig. 5). In nasal samples, dispersal limitation dominated in NS and FS1y, while smoking slightly increased contributions of undominated drift and homogeneous selection, persisting after 6 weeks cessation (FS6w). Oropharyngeal communities were largely shaped by dispersal limitation in NS, AS and FS6w, with undominated drift increasing after long-term cessation. BAL communities were influenced by both stochastic and deterministic processes, with homogeneous selection particularly prominent in AS and, to a lesser extent, in FS6w, while normalizing after 1-year cessation.

**Fig. 6:**
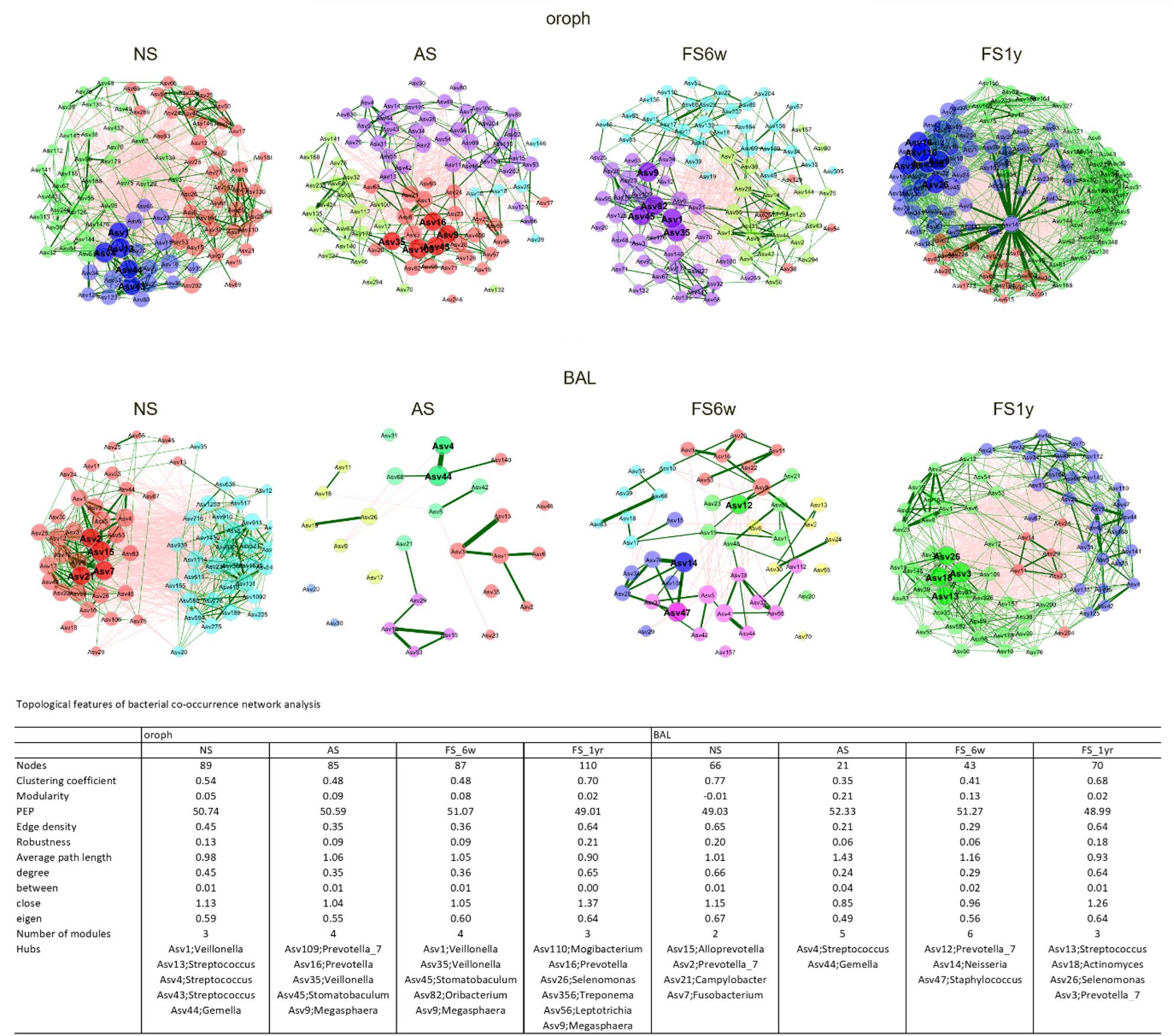
*Microbial co-occurrence patterns *Bacterial co-occurrence network analysis of core ASV (defined as ASV present in at least 50% of subjects) for never- (NS, *n*=10), active (AS, *n*=15) and after smoking cessation (FS6w, *n*=15; FS1y, *n*=5) in oropharynx and BAL. Nodes indicate ASV and edges represent Pearson correlation (threshold 0.3) between a pair of nodes. The green and red links represent positive and negative network interactions, respectively. Modules represent groups of interconnected nodes, each differently colored. Bold nodes represent hub taxa, whereas node sizes indicate their respective eigenvectors. The table shows important topological features of bacterial co-occurrence networks, including number of nodes, clustering coefficient, modularity, positive edge percentage (PEP), edge density, robustness, average path length, centrality measures (degree, between, close, eigenvector) and number of modules.

Microbial network analysis for the oropharynx and BAL revealed pronounced shifts during smoking and cessation (Fig. 6). In oropharynx, smoking reduced network complexity (fewer nodes, lower edge density and degree centrality, reduced robustness) while increasing modularity and path length. Short-term cessation had minimal impact, whereas long-term cessation restored connectivity, robustness, and degree centrality, resulting in denser but less modular networks than in NS. In BAL, smoking induced stronger network reductions, with restructuring evident already at 6 weeks and continuing through 1 year, leading to networks with comparable robustness, edge density, and degree centrality, though modularity remained elevated relative to NS. Due to the low number of core ASV, nasal networks could not be calculated.

In both oropharynx and BAL, pronounced changes were observed in the hub taxa (key microbial species that play central roles in maintaining network stability by exhibiting high levels of connectivity within the microbial community). In the oropharynx of NS, ASV assigned to *Veillonella*, *Streptococcus*, and *Gemella* were identified as central hubs. Smoking shifted the central hubs to ASV related to *Prevotella*, *Veillonella*, *Megasphaera*, and *Stomatobaculum*, mainly persisting in FS6w. After 1-year cessation, *Veillonella* and *Stomatobaculum* were diminished and new hub ASV (e.g. assigned to *Prevotella*,* Selemonas*,* Leptotrichia*) emerged in the oropharynx. In BAL, smoking shifted central hubs from ASV assigned to *Prevotella* spp., *Camylobacter* and *Fusobacterium* in NS towards *Streptococcus*- and *Gemella*-related ASV. In FS6w, BAL hubs included ASV assigned to *Prevotella_7* and *Neisseria*, followed by *Prevotella_7*,* Actinomyces*,* Selemonas* and *Streptococcus* after 1 year cessation.

In summary, microbial community assembly was predominantly stochastic in the URT, whereas the LRT showed a substantial deterministic contribution, and although cessation led to a gradual recovery of network structure, this was accompanied by persistent shifts in central ASV.

## Discussion

Understanding how smoking alters the respiratory microbiome and whether these changes are reversible is critical for developing interventions to restore airway health. The longitudinal, multi- compartment design of our study demonstrates that smoking induces site-specific alterations in the respiratory microbiome, with increased individuality in the nasal, oropharyngeal, and lung communities. In our cohort, short-term cessation was associated with partial restoration of richness and community composition, particularly in the lower airways, whereas long-term cessation in the small subgroup showed incomplete recovery and persistent inter-individual variation. Recovery trajectories were highly individualized, reflecting both stochastic and deterministic ecological processes and highlight the pronounced impact of smoking on respiratory microbial networks.

### Microbial communities among the respiratory tract are niche-specific

Oropharyngeal and BAL microbiomes overlapped in NS and were dominated by *Actinomyces*,* Prevotella* groups, *Streptococcus* and *Veillonella*, whereas the nose was dominated by *Corynebacterium* and *Staphylococcus*. This reflects microaspiration and aligns with previous reports showing that the lung microbiome resembles the oropharynx, whereas the nose is more similar to the skin [14, 32]. Each site hosts a unique, dynamic community shaped by airflow, mucociliary clearance, immune defences, and local microenvironments [10, 33].

### The effect of smoking and cessation on the microbiome is highly dependent on the airway compartment

Smoking alters airway microbiomes via oxygen depletion, antimicrobial activity, biofilm promotion, and immune impairment, enriching potential pathogens such as *Streptococcus pneumoniae* and *Haemophilus influenza* [24, 34]. In our study, both genera remained elevated after cessation, linking smoking to COPD risk [35, 36]. In line with reports from oral and gut microbiome studies [37, 38], smoking enriched strict anaerobic bacteria such as *Prevotella* and *Veillonella*, which are known to modulate immunity and inhibit pathogens but may also increase susceptibility to inflammatory diseases if dysregulated [8, 39]. Notably, *Prevotella* spp. responses were heterogeneous, with distinct dynamics observed across subgroups (e.g., *Prevotella* vs. *Prevotella_7*), likely reflecting intra-genus variability and differences in oxygen tolerance among clades.

In the oropharynx, *Neisseria* decreased with smoking as previously reported [14], likely due to oxygen depletion, but recovered rapidly post-cessation. It has been reported that *Neisseria* species can grow in anaerobic biofilms exhibiting denitrification [40, 41] and, by this, produce nitrite and possibly nitric oxide, both of which may act as antimicrobial compounds. This may explain the fast re-establishment of *Neisseria* observed after cessation. Despite high overlap in shared genera and substantial resilience, oropharyngeal communities in this small long-term subgroup did not fully revert to those of NS after 1 year. Long-term cessation favoured colonization by biofilm-forming oral bacteria often associated with periodontitis like *Aggregatibacter*,* Dialister* and *Fretibacterium* [42–44], occupying niches freed by smoking-induced disturbance.

Smoking effects were even more pronounced in the lung. While bacterial diversity appeared to recover after 1 year, community composition in our subgroup remained altered. In line with reports from gut microbiome studies [45, 46], *Alistipes*, Muribaculaceae, and Prevotellaceae were depleted, likely disrupting immune and metabolic homeostasis. Oral- and gut-associated biofilm-forming genera (e.g. *Capnocytophaga*,* Granulicatella*,* Parvimonas*) colonized the lung after smoking cessation, reflecting URT-LRT microbial exchange and re-shaping of communities towards greater oropharynx-lung similarity. Our longitudinal data suggest that smoking imposes lasting effects on airway microbiomes, with partial recovery over time, consistent with previous reports [25]. However, results must be interpreted with caution due to the small sample size at the one-year time point and the potential limitations in generalizing results to a broader population.

### Smoking favours individual variability long-lasting as result of site-specific assembly processes

Smoking increased inter-individual variability across all airway sites, which persisted after cessation. In the nose, microbial community assembly was largely driven by stochastic processes, likely amplified by high environmental exposure (fluctuating temperature, humidity and airborne particles) and smoke-induced host effects, explaining the high degree of personalization. In contrast, the oropharyngeal microbiome, buffered by saliva [47], maintained a relatively stable core community but showed increased inter-individual variability associated with smoking duration and intensity, reflecting priority effects and niche colonization. Lung communities were strongly shaped by deterministic processes, consistent with its low microbial load, restricted immigration, and higher immune surveillance [32, 48, 49]; smoking intensified host-driven selection, favouring specific taxa. Partial recovery post-cessation indicates that deterministic and stochastic forces jointly drive long-term individual patterns. These findings may help to explain the heterogeneity of smoking-related airway diseases [50–52]: stochastic colonization in the upper airways could promote variable susceptibility to infections such as rhinitis or sinusitis, whereas deterministic enrichment of smoke-tolerant taxa in the lung may drive more consistent risks for COPD or asthma exacerbations.

Although partial microbial recovery after cessation may indicate some potential for therapeutic intervention, persistent alterations and highly personalized responses highlight challenges in developing broadly applicable strategies. Preclinical and clinical studies suggest that dietary fiber, probiotics (*Lactobacillus* spp., *Bifidobacterium* spp.), and fecal microbiota transplantation can modulate immune responses and influence respiratory outcomes [53–56] and restore microbial gut communities after disturbance [57], providing proof-of-principle for potential therapeutic concepts. For the nose and oropharynx, approaches enhancing competitive colonization or microbial reseeding may counteract stochastic divergence, whereas in the lung therapies focusing on restoring the host environment and selective pressures may be more effective. However, developing targeted interventions to accelerate microbial recovery post-cessation requires a deeper understanding of the compartment-specific balance between stochastic and deterministic forces driving the individual smoking-related changes and recovery.

### The effect of smoking and cessation on microbial co-occurrence depends on the airway compartment

Smoking reduced the network connectivity, robustness, and centrality while increasing modularity and path length in oropharynx and BAL, reflecting disrupted microbial interactions. In the oropharynx, networks recovered slowly, eventually forming denser, less modular communities after smoking cessation, indicating reorganization of central taxa and cooperative behaviour. In the oropharynx of NS, hubs were assigned to *Veillonella*, *Streptococcus*, and *Gemella*. *Streptococcus* and *Gemella* act as early colonizers, metabolizing host-derived carbohydrates and initiating biofilms, while *Veillonella* consumes streptococcal lactate, exemplifying metabolic cross-feeding that stabilizes microbial networks [58, 59]. Together, these hubs support a balanced ecosystem structured around carbohydrate metabolism, niche complementarity, and controlled biofilm development promoting mucosal homeostasis. Smoking shifted hubs towards ASV related to *Prevotella* and *Megasphaera*, strict anaerobic bacteria capable of degrading complex carbohydrates and amino acids into short-chain fatty acids (SCFA) [60, 61] and modulating mucosal immune responses [56, 62]. This suggests a network restructured toward a fermentation-dominated state with altered host–microbe interactions. After cessation, the network did not fully revert to the NS state. While *Prevotella*,* Veilonella* and *Megasphaera* persisted as hubs, new central taxa such as ASV assigned to *Leptotrichia* emerged. *Leptotrichia* spp. are saccharolytic fermenting bacteria producing lactate and other SCFA [63], potentially re-establishing trophic links with lactate-utilizing organisms like *Veillonella*.

In BAL, smoking-induced network reduction was more pronounced than in the oropharynx. However, short-term cessation rapidly improved connectivity and robustness, with further recovery following long-term cessation. BAL-NS hubs included ASV assigned to *Prevotella* spp., and *Fusobacterium*, genera common in healthy lower airways and thought to originate from microaspiration of oral communities [32]. While *Prevotella* spp. can modulate immune responses via SCFA production, *Fusobacterium* contributes to biofilm coaggregation [59, 62]. Smoking shifted hubs towards ASV related to *Streptococcus* and *Gemella*, reflecting expansion of URT-origin genera normally limited by low biomass and/or host defenses. Following cessation, *Prevotella_7* established as a hub in BAL, indicating partial recovery of a healthy lower airway community and restoration of SCFA-mediated immune modulation [32, 62]. New hubs emerged, including ASV assigned to *Streptococcus* and *Selenomonas*, reflecting network reorganization and metabolic cross-feeding with *Selemonas* being capable of consuming streptococcal lactate and other fermentation products [60].

Collectively, these patterns suggest ecological succession rather than simple reversal. Smoking altered central taxa and metabolic coupling, and cessation promoted reorganization toward a mature, anaerobic biofilm community that was functionally distinct from the original NS state. Differences between the oropharynx and BAL reflect ecological contrasts: high-dispersal, stochastic environments in the oropharynx versus low-biomass, deterministic filtering in the lung, shaping both the intensity and speed of recovery. However, long-term cessation results must be interpreted with caution due to the small sample size (*n* = 5).

### Limitations of the study

Although the longitudinal, multi-compartment design allows to track microbial changes over time, the relatively small sample size after 1-year cessation limits broad conclusions. Furthermore, the mono-centric design may restrict the generalizability of these findings to larger and/or more diverse populations. In addition, this study exclusively included conventional cigarette smokers; therefore, the findings cannot be directly extrapolated to users of alternative nicotine delivery systems such as e-cigarettes, which may exert other effects on the respiratory microbiome. These factors warrant caution when interpreting the findings, particularly regarding long-term and product-specific effects of smoking cessation on the respiratory microbiome. Additionally, the 16S rRNA gene–based metabarcoding approach provides valuable insights into community composition; however, the use of short-read sequencing (~ 300 bp) limited taxonomic resolution predominantly to the genus level. As a result, species- or strain-level differences, often associated with variation in ecological function and pathogenic potential, could not be resolved. Moreover, the marker gene approach does not allow direct assessment of functional attributes or metabolic activity, which would be necessary to better understand microbial interactions. Thus, further longitudinal studies including long-read metagenome sequencing in combination with metabolomics are warranted to characterize functional changes in the of microbial responders and the overall community. Such studies should ideally adopt multi-centric designs to account for population heterogeneity.

## Conclusion

This study demonstrates that smoking induces site-specific alterations in the respiratory microbiome, with partial recovery after cessation, particularly in the lower airways. In our cohort, microbial inter-individual variability remained increased after cessation, reflecting a complex interplay of stochastic and deterministic processes. These findings underscore the importance of considering microbial dynamics across the entire respiratory tract when studying the effects of smoking and cessation. Nonetheless, the limited number of subjects 1-year after cessation warrants cautious interpretation. Future studies incorporating larger, multi-center longitudinal cohorts and functional genomic approaches will be essential to validate our findings, further elucidate the mechanisms underlying microbial recovery following smoking cessation, and clarify their potential clinical implications. 

## Supplementary Information

Supplementary Material 1.

Supplementary Material 2.

Supplementary Material 3.

## Data Availability

The datasets generated and analyzed during the current study have been deposited in the NCBI Sequence Read Archive (SRA) under BioProject accession PRJNA1328433. R scripts, abundance tables and minimal, non-identifiable sample metadata (sample ID, respiratory site, and smoking status) used in this study are publicly available via Zenodo at [https://doi.org/10.5281/zenodo.18889166].

## Abbreviations

AS: active smokers
ASV: amplicon sequence variants
BAL: bronchoalveolar lavage
COPD: chronic obstructive pulmonary disease
cpd: cigarettes per day
cpd_max: maximum cigarettes per day
CRS: chronic rhinosinusitis
FS: former smokers
FS1y: former smokers after 1 year cessation
FS6w: former smokers after 6 weeks cessation
GLMM: generalized linear mixed-effect model
LRT: lower respiratory tract
NMDS: non-metric multidimensional scaling
NS: never-smokers
RT: respiratory tract
SCFA: short chain fatty acids
URT: upper respiratory tract
