# Supplementary material for: Longitudinal dynamics and site-specific recovery of the human respiratory microbiome following smoking cessation
Source: Respir Res. 2026 Apr 2;27:163. doi: 10.1186/s12931-026-03644-z (PMC13064275; doi:10.1186/s12931-026-03644-z)
Supplement: Supplementary file 1 — Supplementary Material 1. [file 12931_2026_3644_MOESM1_ESM.docx]

**Additional Information: Materials and Methods**

*DNA extraction*

The cell pellet of 5 mL BAL (centrifuged at 20.000 x g/10 min) was dissolved in 180 µl lysozyme solution (10mg/µl), whereas nasal and oropharyngeal swabs were covered directly with 400 µl lysozyme solution (10mg/µl). After incubation for 45 min at 37°C, DNA extraction was performed using the PureLink™ Genomic DNA Mini Kit (ThermoFisher Scientific, Altham, USA) according to the manufacturer’s protocol for Gram-positive bacteria. To identify contaminants deriving from sampling and extraction kit, controls were included (DNA extraction of 5 mL sterile saline solution (8 samples) and 5 mL bronchoscope flushing (8 samples), as well as 5 blank extractions).

*16S rRNA gene library preparation*

Amplicon sequencing of the V4 hypervariable region of the 16S rRNA gene was performed on a MiSeq Illumina instrument (MiSeq Reagent Kit v3 (600 Cycle); Illumina, San Diego, CA, USA) using the universal eubacterial primers 515F ^(1)^ and 806R ^(2)^. PCR was done using NEBNext high fidelity polymerase (New England Biolabs, Ipswich, USA) in a total volume of 25 µl (10 ng DNA template, 12.5 µl polymerase, 5 pmol of each primer, 2.5 µl 3% BSA) and the following PCR conditions: 5 min at 98˚C; 30 cycles of 10 s at 98˚C, 30 s at 55 ˚C, 30 s at 72 ˚C; 5 min 72 ˚C. To identify potential contaminants deriving from library preparation, we included 4 blank PCR controls. Subsequent library preparation was performed as described previously ^(3)^.

The sequence data obtained in this study are deposited in the short read archive of NCBI under accession number PRJNA1328433 (reviewer link: https://dataview.ncbi.nlm.nih.gov/object/PRJNA1328433?reviewer=ocn4eho45d9g7t8f6u07e6tj5m).

*Sequence processing*

FASTQ files were trimmed with a minimum read length of 50 using Cutadapt ^(4)^ and quality control was performed via FastQC ^(5)^. For subsequent data analysis, the DADA2 pipeline v 1.30 ^(6)^ was used with the following trimming and filtering parameters: 20 bp were removed n-terminally and reads were truncated at position 260 (forward) and 200 (reverse), respectively, with expected error of 4 (forward) and 6 (reverse). Taxonomic analysis was performed using SILVA v138.1. Reads were excluded if classified as mitochondria or chloroplast or if the phylum was missing. All blank samples (saline solution, bronchoscope flushing, blank extraction and PCR no template control) were analyzed together with biological samples and showed clearly lower richness and different beta diversity compared to respiratory tract samples (Additional Figure 1). Sequences present in at least 50% of blanks were considered as core contaminome. Additionally, potential contaminants were identified statistically via the ‘decontam’ R package ^(7)^. All sequences of the core contaminome and/or being identified by ‘decontam’ were removed from sample data (in total 45, Additional Table 1), resulting in a total amount of 5,446,191 reads (corresponding to an average of 43,223 reads per sample) assigned to 2,221 amplicon sequence variants (ASV).

*Statistical analysis*

All plots and statistics were performed in R version 4.4.0 (https://www.R-project.org). Sequencing data were normalized using cumulative-sum scaling (CSS) via the metagenomeSeq R package ^(8)^. CSS calculates scaling factors based on the cumulative sum of gene abundances up to a dataset-specific threshold, allowing an accurate comparison of microbial communities across different samples with varying sizes. Prior to the main analysis, potential covariates (age, gender, weight, medical history, pet contact, alcohol consumption, and pollutant exposure) were tested individually for association with microbiome outcomes due to the modest sample size (n = 25). Covariates improving model fit, as judged by metrics like delta AIC, beta coefficients and CI for mixed-effect models or explained variance for PERMANOVA, were included in the final analyses. Results of pre-tests were shown in Additional Table S3.

Alpha diversity was calculated using species richness and evenness. Associations with covariates were assessed using generalized linear mixed-effects models (R package glmmTMB), including study subjects as a random effect to account for repeated measures. Covariates improving model fit in pre-tests (age, alcohol for richness; no covariate for evennes) were included. Beta diversity was analysed via unweighted and weighted UniFrac distance as well as Bray-Curtis dissimilarity using PERMANOVA and strata to restrict permutations within study subjects (R package vegan) Based on pre-tests, only age was included as covariate. For both alpha and beta diversity analysis, p value adjustment for multiple comparisons was performed with Benjamini-Hochberg correction.

To identify microbial taxa differing between never-smokers (NS), active smokers (AS) and former smokers (FS), DESeq2 with age as covariable was used ^(9)^. Taxa were considered as enriched or depleted with a log2-fold change >=2 and an FDR-adjusted p-value < 0.05.

To assess inter-individual variability, a metric quantifying the degree of individuality of each sample was calculated as follows: Bray-Curtis dissimilarities were computed between all pairs of samples. For each sample, the mean distance to other samples from the same individual (within-individual) and to samples from different individuals (between-individual) was determined. The degree of individuality was defined as the difference between these two values, with higher scores reflecting greater uniqueness of a sample relative to other samples from the same individual versus the overall population. Scores range from 0 to 1, where 0 indicates no individuality (the sample is similar to others) and 1 indicates maximal individuality (the sample is highly distinct from others). Based on pre-tests, age was included as covariate in the final generalized linear mixed-effects models (R package glmmTMB), with study subject as a random effect and Benjamini-Hochberg p value correction.

In addition, Bray-Curtis distances were used to assess the recovery potential after cessation by computing the similarity of the microbial community of former smokers per subject towards the NS community. Like for inter-individuality, generalized linear mixed-effects models (R package glmmTMB) with age as a fixed-effect covariate and study subjects as a random effect to account for repeated measurements were applied. P-values were adjusted for multiple testing using the Benjamini-Hochberg procedure.

Furthermore, the similarity of oropharynx and BAL microbial communities within each subject was computed via between-group distances calculated by weighted UniFrac distance measures. Statistical analysis was performed using linear mixed-effects models including the study subjects (longitudinal samples) as repeated measurements by defining a random effect term (R package lme4) and P value adjustment for multiple comparisons with Benjamini-Hochberg correction.

To search for correlations between smoking-related factors (smoking years, cigarettes per day (cpd), packyears and exposure based on nicotine levels) and the degree of individual variability or recovery, Pearson correlations were calculated, using Benjamini-Hochberg p value adjustment for multiple pairwise comparisons. To find correlations between numeric variables and microbiome data, Spearman correlations were calculated between clr-transformed abundance data and meta variables, using Benjamini-Hochberg p value adjustment for multiple pairwise comparisons.

The core microbiome per group (respiratory cavity, smoking status) was defined as genera present in at least 80 % of subjects without setting an additional abundance cutoff.

For the determination of microbial assembly processes, the ßNTI and Raup-Crick-based Bray-Curtis (RCbray) method described by Stegen et al. ^(10)^ was used. Although βNTI was originally developed for macro-ecological systems, it has been widely adopted in microbial ecology because it quantifies phylogenetic turnover relative to null expectations, allowing inference of deterministic versus stochastic assembly processes. In microbial communities, phylogenetic relatedness is a reasonable proxy for ecological similarity due to phylogenetic signals in key traits. This framework was applied to our dataset to disentangle deterministic and stochastic processes shaping microbial communities across smoking groups and respiratory sites, where smoking may act as a strong selective pressure. For βNTI calculations, thresholds of ±2 indicate deterministic selection, distinguished into heterogeneous/variable selection (selective pressures drive communities to divergent configurations; ßNTI > +2) and homogeneous selection (selective pressures push communities towards a common composition; ßNTI < -2). For pairwise comparisons with |βNTI| < 2, RCbray was used to distinguish stochastic processes using classical cutoffs: RCbray > +0.95 for dispersal limitation (populations are unable to mix, leading to development via ecological drift), RCbray < -0.95 for homogenizing dispersal (communities are more similar than expected; populations are capable of interactions, allowing members to freely exchange), and |RCbray| ≤ 0.95 for undominated processes (no assembly process is capable of explaining variation). βNTI and RCbray were calculated in R using the packages picante and vegan. Null distributions were generated using 999 randomizations. A phylogenetic tree was constructed prior to these analyses by first aligning sequences with MAFFT (FFT-NS algorithm) and then generating a tree with FastTree under the GTR+CAT nucleotide substitution model. The R package ape was used to handle phylogenetic distances via a phyloseq object.

Microbial co-occurrence networks were inferred via SparCC correlations implemented in the R package NetCoMi v1.1.0 ^(11)^, using the “signed” transformation to transform the estimated associations into dissimilarities and including only ASV present in 50% of the samples per group. Highly interconnected modules of nodes within the networks were then identified using the cluster_fast_greedy algorithm.

All plots were created in R using ggplot2 ^(12)^, ggpubr ^(13)^, and eulerr ^(14)^.

Supplementary References

1. Apprill A, McNally S, Parsons R, Weber L. Minor revision to V4 region SSU rRNA 806R gene primer greatly increases detection of SAR11 bacterioplankton. Aquat Microb Ecol. 2015;75.

2. Parada AE, Needham DM, Fuhrman JA. Every base matters: assessing small subunit rRNA primers for marine microbiomes with mock communities, time series and global field samples. Environ Microbiol. 2016;18(5):1403-14.

3. Musiol S, Harris CP, Gschwendtner S, Burrell A, Amar Y, Schnautz B, et al. The impact of high-salt diet on asthma in humans and mice: Effect on specific T-cell signatures and microbiome. Allergy. 2024;79(7):1844-57.

4. Martin M. Cutadapt removes adapter sequences from high-throughput sequencing reads. EMBnetjournal. 2011;17:10-2.

5. Andrews S. FastQC: A Quality Control Tool for High Throughput Sequence Data. https://wwwbioinformaticsbabrahamacuk/projects/fastqc/. 2010.

6. Callahan BJ, McMurdie PJ, Rosen MJ, Han AW, Johnson AJ, Holmes SP. DADA2: High-resolution sample inference from Illumina amplicon data. Nat Methods. 2016;13(7):581-3.

7. Davis NM, Proctor DM, Holmes SP, Relman DA, Callahan BJ. Simple statistical identification and removal of contaminant sequences in marker-gene and metagenomics data. Microbiome. 2018;6(1):226.

8. Paulson JN, Stine OC, Bravo HC, Pop M. Differential abundance analysis for microbial marker-gene surveys. Nat Methods. 2013;10(12):1200-2.

9. Love MI, Huber W, Anders S. Moderated estimation of fold change and dispersion for RNA-seq data with DESeq2. Genome Biol. 2014;15(12):550.

10. Stegen JC, Lin X, Fredrickson JK, Chen X, Kennedy DW, Murray CJ, et al. Quantifying community assembly processes and identifying features that impose them. ISME J. 2013;7(11):2069-79.

11. Peschel S. NetCoMi: Network Construction and Comparison for Microbiome Data. https://netcomide. 2025;R package version 1.2.0.

12. Wickham H. ggplot2: Elegant Graphics for Data Analysis. https://ggplot2tidyverseorg. 2016.

13. Kassambara A. ggpubr: 'ggplot2' Based Publication Ready Plots. R package version 0.6.0. https://rpkgsdatanoviacom/ggpubr/. 2023.

14. Larsson JG, Peter. A Case Study in Fitting Area-Proportional Euler Diagrams with Ellipses using eulerr. Proceedings of International Workshop on Set Visualization and Reasoning (SetVR 2018) co-located with 10th International Conference on the Theory and Application of Diagrams (Diagrams 2018), Edinburgh, UK, June 18, 2018 (pp 84-91). 2018;CEUR-WS.org. https://ceur-ws.org/Vol-2116/paper7.pdf.
