# Supplementary material for: Longitudinal dynamics and site-specific recovery of the human respiratory microbiome following smoking cessation
Source: Respir Res. 2026 Apr 2;27:163. doi: 10.1186/s12931-026-03644-z (PMC13064275; doi:10.1186/s12931-026-03644-z)
Supplement: Supplementary file 2 — Supplementary Material 2. [file 12931_2026_3644_MOESM2_ESM.pdf]

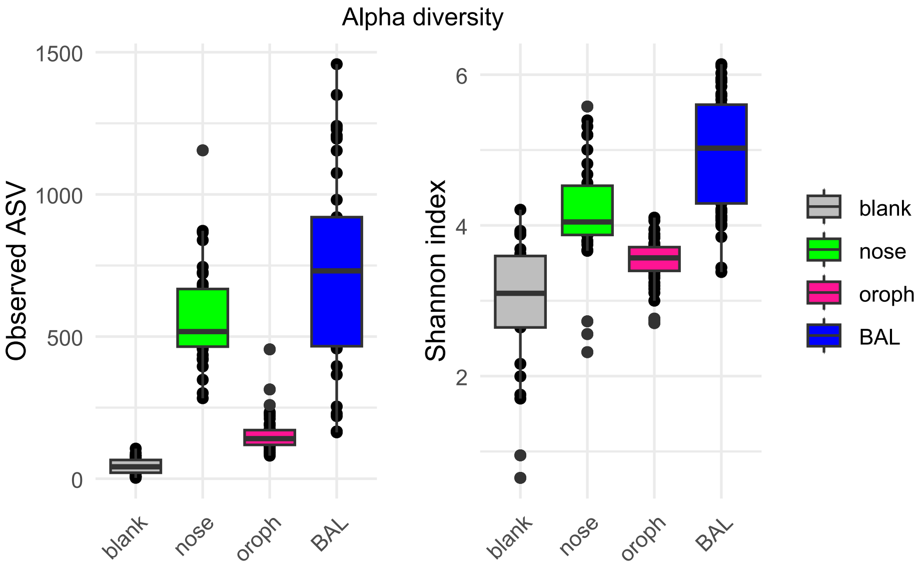

	term	estimate	std.error	statistic	p.value
Observed	(Intercept)	3.807682653	0.109891625	34.64943431	4.56E-263
	nose	2.488888568	0.148285531	16.78443308	3.17E-63
	oroph	1.210470536	0.136272959	8.882690629	6.53E-19
	BAL	2.739651939	0.141450308	19.3682996	1.43E-83
Shannon	(Intercept)	1.075727817	0.116798859	9.210088432	3.26E-20
	nose	0.356782214	0.142382513	2.505800798	0.012217443
	oroph	0.182886381	0.141259102	1.294687411	0.195428085
	BAL	0.527020795	0.134596602	3.915557951	9.02E-05

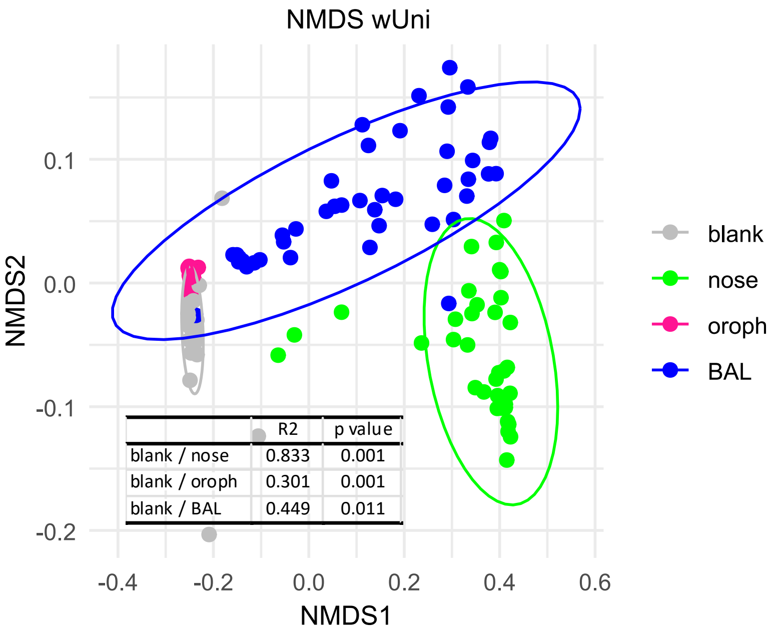

	R2	p value
blank / nose	0.833	0.001
blank / oroph	0.301	0.001
blank / BAL	0.449	0.011

Additional Figure 1. Respiratory tract samples showed a significantly higher alpha diversity and different community composition compared to blank samples.

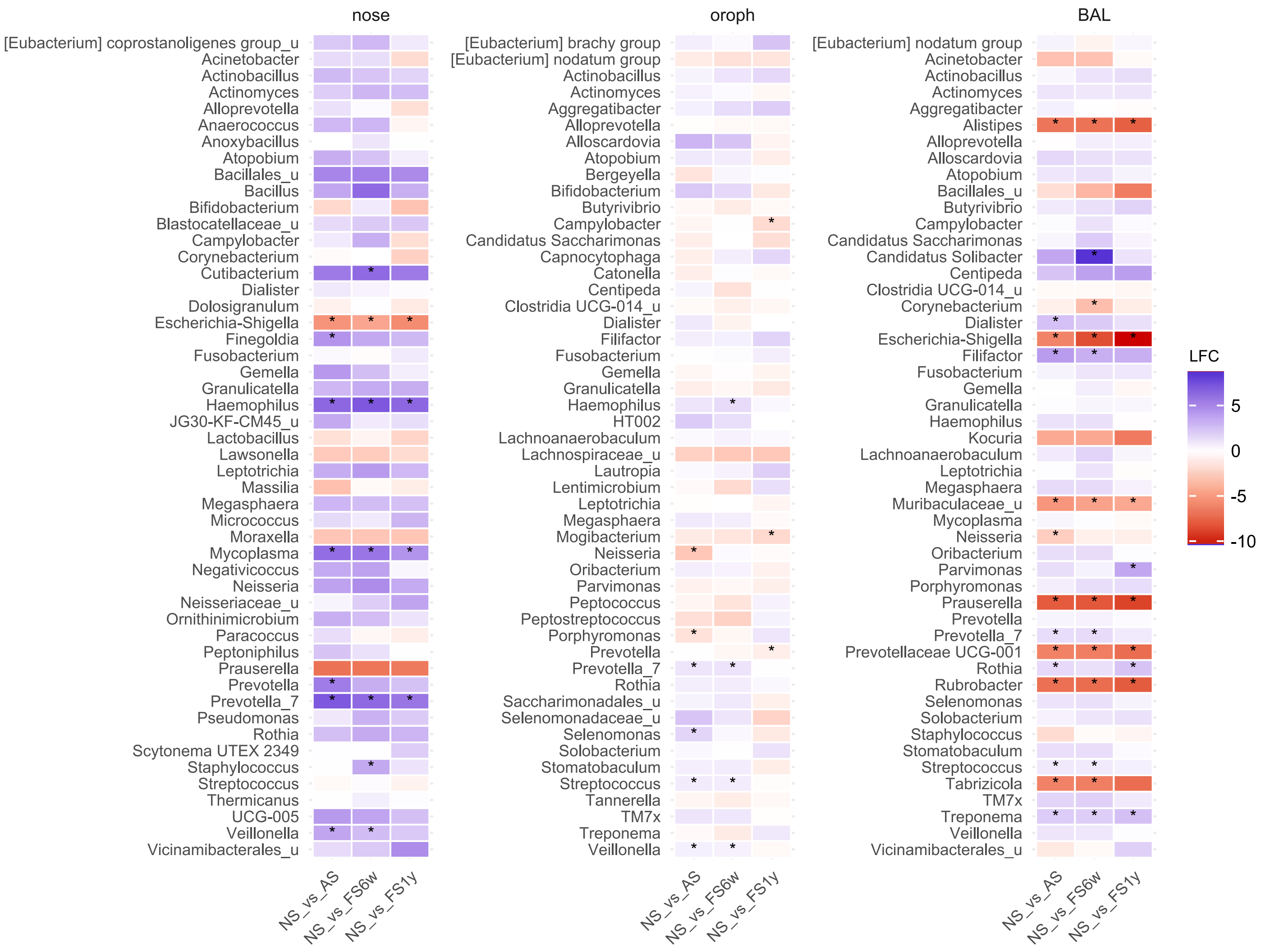

Additional Figure 2. Heatmaps show changes of the top 15 taxa in nose, oropharynx and BAL for the active smokers (AS), former smokers 6 weeks post-cessation (FS6w), and 1-year post-cessation (FS1y), calculated as log-fold changes to never-smokers baseline (NS). Asterisks mark significant differences (p<0.05).

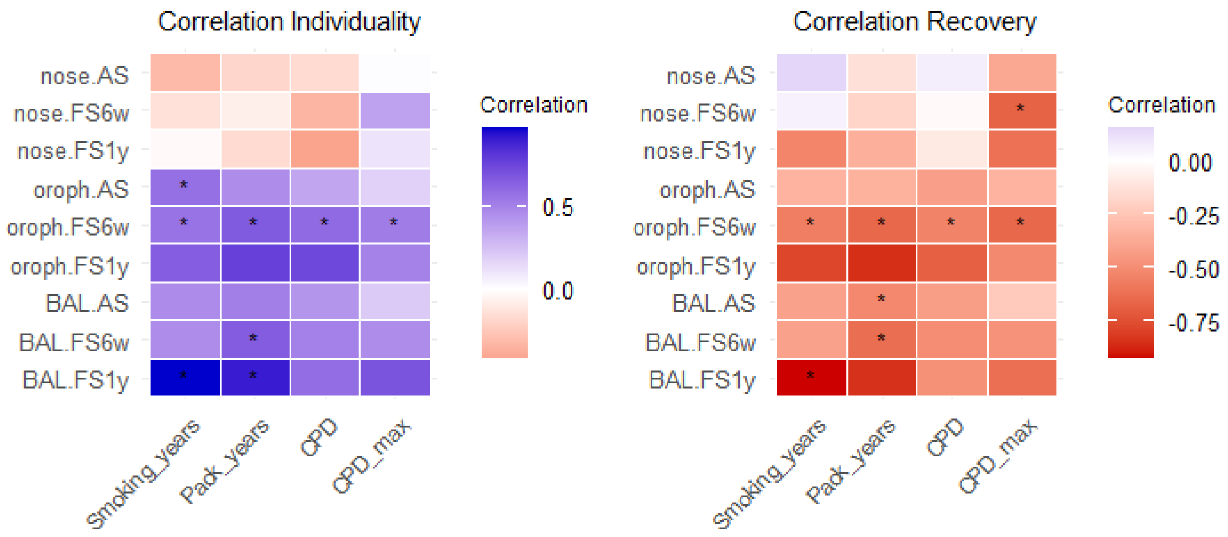

Additional Figure 3. Correlations of inter-individual variability and recovery of microbial community with smoking history (smoking years, packyears, cigarettes per day (CPD) and maximum cigarettes per day (CPD_max) for nose, oropharynx and BAL in active smokers (AS), former smokers 6 weeks post-cessation (FS6w), and 1-year post-cessation (FS1y). Asterisks mark significant differences ($p<0.05$).

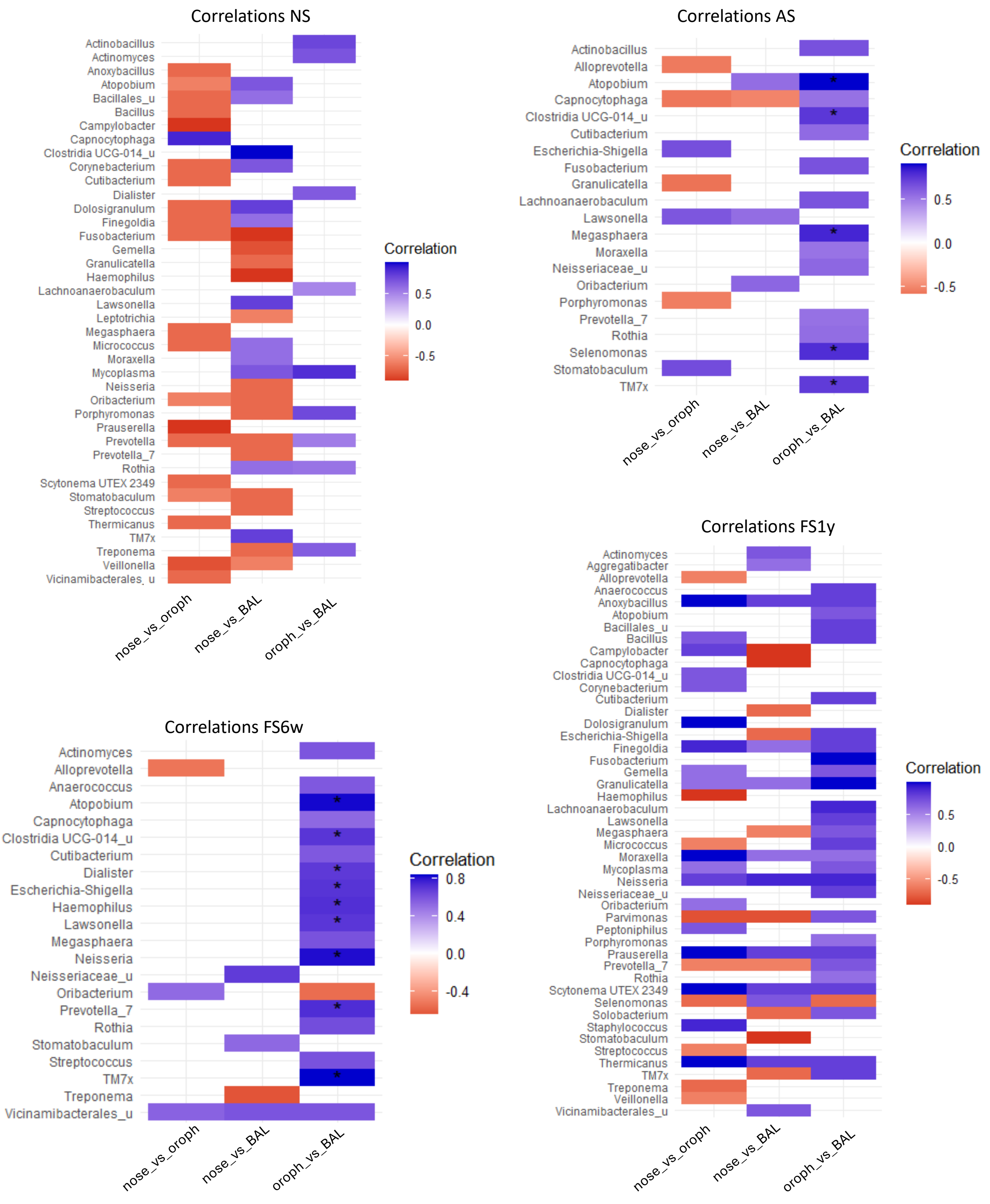

Additional Figure 4. Correlations of taxa between the different respiratory sites in never-smokers (NS), active smokers (AS), former smokers 6 weeks post-cessation (FS6w), and 1-year post-cessation (FS1y). Shown are all correlations > 0.5. Asterisks mark significant differences (p<0.05).
