# Supplementary material for: Longitudinal dynamics and site-specific recovery of the human respiratory microbiome following smoking cessation
Source: Respir Res. 2026 Apr 2;27:163. doi: 10.1186/s12931-026-03644-z (PMC13064275; doi:10.1186/s12931-026-03644-z)
Supplement: Supplementary file 3 — Supplementary Material 3. [file 12931_2026_3644_MOESM3_ESM.pdf]

Additional Table 1. ASV identified as contaminants by core analysis and/or decontam were removed from data.

ASV	phylum	class	order	family	genus
Asv1927	Crenarchaeota	Nitrososphaeria	Nitrososphaerales	*Nitrososphaeraceae*	*Candidatus Nitrocosmicus*
Asv1407	Crenarchaeota	Nitrososphaeria	Nitrososphaerales	*Nitrososphaeraceae*	*unclassified*
Asv1831	Acidobacteriota	Blastocatellia	Pyrinomonadales	*Pyrinomonadaceae*	*RB41*
Asv5265	Acidobacteriota	Subgroup 5	unclassified	*unclassified*	*unclassified*
Asv3083	Acidobacteriota	Vicinamibacteria	Vicinamibacterales	*unclassified*	*unclassified*
Asv2838	Acidobacteriota	Vicinamibacteria	Vicinamibacterales	*Vicinamibacteraceae*	*unclassified*
Asv2098	Actinobacteriota	Actinobacteria	Corynebacteriales	*Corynebacteriaceae*	*Corynebacterium*
Asv1776	Actinobacteriota	Actinobacteria	Corynebacteriales	*Corynebacteriaceae*	*Corynebacterium*
Asv5572	Actinobacteriota	Actinobacteria	FFCH16263	*unclassified*	*unclassified*
Asv4632	Actinobacteriota	Actinobacteria	Frankiales	*Geodermatophilaceae*	*Blastococcus*
Asv3041	Actinobacteriota	Actinobacteria	Micrococcales	*Brevibacteriaceae*	*Brevibacterium*
Asv4298	Actinobacteriota	Actinobacteria	Micrococcales	*Dermabacteraceae*	*Brachybacterium*
Asv1227	Actinobacteriota	Actinobacteria	Micrococcales	*Micrococcaceae*	*Arthrobacter*
Asv73	Actinobacteriota	Actinobacteria	Propionibacteriales	*Propionibacteriaceae*	*Cutibacterium*
Asv5279	Actinobacteriota	Thermoleophilia	Gaiellales	*Gaiellaceae*	*Gaiella*
Asv6857	Actinobacteriota	Thermoleophilia	Gaiellales	*Gaiellaceae*	*Gaiella*
Asv4628	Actinobacteriota	Thermoleophilia	Gaiellales	*unclassified*	*unclassified*
Asv4061	Actinobacteriota	Thermoleophilia	Solirubrobacterales	*67-14*	*unclassified*
Asv5579	Actinobacteriota	Thermoleophilia	unclassified	*unclassified*	*unclassified*
Asv5557	Bacteroidota	Bacteroidia	Cytophagales	*Hymenobacteraceae*	*Adhaeribacter*
Asv6385	Chloroflexi	Anaerolineae	RBG-13-54-9	*unclassified*	*unclassified*
Asv2081	Chloroflexi	KD4-96	unclassified	*unclassified*	*unclassified*
Asv6900	Chloroflexi	KD4-96	unclassified	*unclassified*	*unclassified*
Asv3496	Deinococcota	Deinococci	Deinococcales	*Deinococcaceae*	*Deinococcus*
Asv7500	Desulfobacterota	unclassified	unclassified	*unclassified*	*unclassified*
Asv1161	Firmicutes	Bacilli	Staphylococcales	*Staphylococcaceae*	*Staphylococcus*
Asv5561	Gemmatimonadota	Gemmatimonadetes	Gemmatimonadales	*Gemmatimonadaceae*	*unclassified*
Asv2075	Methylomirabilota	Methylomirabilia	Rokubacteriales	*unclassified*	*unclassified*
Asv2311	Methylomirabilota	Methylomirabilia	Rokubacteriales	*unclassified*	*unclassified*
Asv7409	Myxococcota	Polyangia	Polyangiales	*BIrii41*	*unclassified*
Asv5537	Proteobacteria	Alphaproteobacteria	Caulobacterales	*Caulobacteraceae*	*Caulobacter*
Asv3427	Proteobacteria	Alphaproteobacteria	Rhizobiales	*Beijerinckiaceae*	*Methylobacterium*
Asv2058	Proteobacteria	Alphaproteobacteria	Rhizobiales	*Xanthobacteraceae*	*Bradyrhizobium*
Asv3569	Proteobacteria	Alphaproteobacteria	Sphingomonadales	*Sphingomonadaceae*	*Ellin6055*
Asv5901	Proteobacteria	Alphaproteobacteria	Sphingomonadales	*Sphingomonadaceae*	*Sphingomonas*
Asv3190	Proteobacteria	Gammaproteobacteria	Incertae Sedis	*Unknown Family*	*Acidibacter*
Asv3033	Proteobacteria	Gammaproteobacteria	PLTA13	*unclassified*	*unclassified*
Asv365	Proteobacteria	Gammaproteobacteria	Pseudomonadales	*Moraxellaceae*	*Acinetobacter*
Asv542	Proteobacteria	Gammaproteobacteria	Pseudomonadales	*Moraxellaceae*	*Acinetobacter*
Asv737	Proteobacteria	Gammaproteobacteria	Pseudomonadales	*Moraxellaceae*	*Enhydrobacter*
Asv933	Proteobacteria	Gammaproteobacteria	Pseudomonadales	*Pseudomonadaceae*	*Pseudomonas*
Asv4042	Proteobacteria	Gammaproteobacteria	Pseudomonadales	*Pseudomonadaceae*	*Pseudomonas*
Asv8224	Proteobacteria	Gammaproteobacteria	Steroidobacterales	*Steroidobacteraceae*	*unclassified*
Asv4045	Verrucomicrobiota	Verrucomicrobiae	Chthoniobacterales	*Chthoniobacteraceae*	*Candidatus Udaeobacter*
Asv6762	Verrucomicrobiota	Verrucomicrobiae	Verrucomicrobiales	*Verrucomicrobiaceae*	*Roseimicrobium*

Additional Table 2. Core microbiota defined as genera observed in at least 80% of subjects per material (nose, oropharynx and BAL) and smoking status, shown as presence (1) and absence (0).

	BAL				nose				oroph			
Taxa	NS	AS	FS6w	FS1y	NS	AS	FS6w	FS1y	NS	AS	FS6w	FS1y
[Eubacterium] brachy group	0	0	0	0	0	0	0	0	0	0	0	1
[Eubacterium] nodatum	0	0	0	0	0	0	0	0	1	0	1	1
Acinetobacter	1	0	0	0	0	0	0	0	0	0	0	0
Actinomyces	1	1	1	1	0	0	0	0	1	1	1	1
Aggregatibacter	0	0	0	0	0	0	0	0	0	0	0	1
Alistipes	1	0	0	0	0	0	0	0	0	0	0	0
Alloprevotella	1	1	1	1	0	0	0	0	1	1	1	1
Amaricoccus	1	0	0	0	0	0	0	0	0	0	0	0
Atopobium	1	1	1	1	0	0	0	0	1	1	1	1
Bacteroides	1	0	0	0	0	0	0	0	0	0	0	0
Bergeyella	0	0	0	1	0	0	0	0	1	0	1	1
Bulleidia	0	0	0	0	0	0	0	0	0	0	0	1
Burkholderia	1	0	0	0	0	0	0	0	0	0	0	0
Butyrivibrio	0	0	0	0	0	0	0	0	1	0	0	1
Campylobacter	1	0	1	1	0	0	0	0	1	1	1	1
Candidatus Saccharimonas	0	0	0	0	0	0	0	0	1	1	1	1
Capnocytophaga	0	0	0	1	0	0	0	0	1	1	1	1
Catonella	0	0	0	1	0	0	0	0	1	1	1	1
Clostridia UCG-014_u	1	0	0	1	0	0	0	0	1	1	1	1
Corynebacterium	1	0	0	0	1	1	1	0	0	0	0	0
Dialister	0	0	0	0	0	0	0	0	0	1	1	1
Escherichia-Shigella	1	0	0	0	1	0	0	0	0	0	0	0
F0058	0	0	0	1	0	0	0	0	0	0	0	0
Filifactor	0	0	0	1	0	0	0	0	0	0	0	1
Fretibacterium	0	0	0	0	0	0	0	0	0	0	0	1
Fusobacterium	1	1	1	1	0	0	0	0	1	1	1	1
Gemella	1	1	1	1	0	0	0	0	1	1	1	1
Granulicatella	0	0	1	1	0	0	0	0	1	1	1	1
Haemophilus	1	1	1	1	0	0	0	0	1	1	1	1
Kocuria	1	0	0	0	0	0	0	0	0	0	0	0
Lachnoanaerobaculum	0	0	0	1	0	0	0	0	1	1	1	1
Lachnospiraceae NK4A136	1	0	0	0	0	0	0	0	0	0	0	0
Lautropia	0	0	0	0	0	0	0	0	0	0	0	1
Lawsonella	0	0	0	0	1	0	0	0	0	0	0	0
Lentimicrobium	0	0	0	0	0	0	0	0	0	0	0	1
Leptotrichia	1	1	1	1	0	0	0	0	1	1	1	1
Megasphaera	1	0	1	1	0	0	0	0	1	1	1	1
Mobiluncus	0	0	0	0	0	0	0	0	0	0	0	1
Mogibacterium	0	0	0	1	0	0	0	0	1	1	1	1
Muribaculaceae_u	1	0	0	0	0	0	0	0	0	0	0	0
Mycoplasma	1	0	0	1	0	1	0	0	0	0	0	1
Neisseria	1	0	1	1	0	0	0	0	1	1	1	1
Oribacterium	1	0	1	0	0	0	0	0	1	1	1	1
Parvimonas	0	0	0	1	0	0	0	0	1	1	0	1
Peptococcus	0	0	0	0	0	0	0	0	0	0	0	1
Peptoniphilus	0	0	0	0	0	1	0	0	0	0	0	0
Peptostreptococcus	0	0	0	0	0	0	0	0	0	0	0	1
Porphyromonas	1	1	1	1	0	0	0	0	1	1	1	1
Prauserella	1	0	0	0	1	0	0	0	0	0	0	0
Prevotella	1	1	1	1	0	0	0	0	1	1	1	1
Prevotella_7	1	1	1	1	0	0	0	0	1	1	1	1
Prevotellaceae UCG-001	1	0	0	0	0	0	0	0	0	0	0	0
Rikenellaceae RC9 gut group	0	0	0	0	0	0	0	0	0	0	0	1
Rothia	1	1	1	1	0	0	0	0	1	1	1	1
Rubrobacter	1	0	0	0	0	0	0	0	0	0	0	0
Saccharimonadales_u	0	0	0	0	0	0	0	0	0	0	1	0
Selenomonas	1	0	1	1	0	0	0	0	1	1	1	1
Solobacterium	1	0	1	1	0	0	0	0	1	1	1	1
Staphylococcus	1	0	0	1	1	1	1	1	0	0	0	0
Stomatobaculum	0	0	0	1	0	0	0	0	1	1	1	1
Streptococcus	1	1	1	1	1	1	1	1	1	1	1	1
Tannerella	0	0	0	1	0	0	0	0	1	1	1	1
TM7x	0	0	1	0	0	0	0	0	1	1	1	1
Treponema	1	1	1	1	0	0	0	0	0	1	1	1
Veillonella	1	1	1	1	0	1	0	0	1	1	1	1
Vicinamibacterales_u	0	0	0	1	0	0	0	0	0	0	0	0

Additional Table 3. Results of covariate tests.

	Covariate	Delta_AIC	Beta	CI_low	CI_high	p_value	Included
Observed	Gender	1.65051133	-0.050391	-0.2148945	0.11411255	0.5482462	-
	Age	-2.8102871	-0.0067329	-0.0124408	-0.001025	0.02077934	+
	Weight	1.50887376	-0.001587	-0.0059448	0.00277077	0.47535355	-
	Hayfever	1.8803279	0.03734876	-0.1743874	0.2490849	0.72954551	-
	Allergy	0.45852496	0.1076751	-0.0606799	0.27603015	0.21000189	-
	Pets	1.21872238	-0.0716373	-0.2307071	0.08743251	0.37740448	-
	Pollutants	-1.3734449	-0.198493	-0.4039922	0.00700623	0.05833442	-
	Alcohol	-2.9305472	0.16410574	0.0277202	0.30049128	0.0183555	+
Evenness	Gender	1.08543923	-0.123065	-0.3700469	0.12391683	0.32875725	-
	Age	1.69927913	0.0026756	-0.0068423	0.01219352	0.58164797	-
	Weight	1.81795847	0.00150578	-0.0053939	0.00840546	0.668835	-
	Hayfever	1.194617	-0.1499323	-0.4756184	0.17575369	0.36689611	-
	Allergy	-0.1772034	-0.1975218	-0.4589839	0.0639403	0.13869083	-
	Pets	1.9488199	-0.0284375	-0.2745565	0.21768146	0.8208392	-
	Pollutants	1.86308442	-0.0608407	-0.3821217	0.26044031	0.71051584	-
	Alcohol	1.1492504	-0.1061565	-0.3301998	0.11788685	0.35305004	-
Individuality	Gender	1.1326153	-0.0774277	-0.2389841	0.0841288	0.34755062	-
	Age	-2.5205461	0.00654587	0.00091931	0.01217244	0.02259357	+
	Weight	0.43859398	-0.0028733	-0.0072494	0.00150285	0.19813056	-
	Hayfever	1.82661871	-0.0440775	-0.2506686	0.16251359	0.67581675	-
	Allergy	1.97025283	-0.0148318	-0.1831049	0.15344134	0.86284255	-
	Pets	1.3227802	0.06621175	-0.0903875	0.22281105	0.40726987	-
	Pollutants	0.44839493	0.13978092	-0.0737866	0.3533484	0.19955191	-
	Alcohol	1.62331699	-0.045942	-0.191468	0.09958413	0.53607215	-
Recovery	Gender	1.26029813	0.1423216	-0.1796002	0.46424341	0.38620736	-
	Age	-0.1514043	-0.0091594	-0.0208221	0.00250328	0.1237298	+
	Weight	1.40007473	0.00310566	-0.0046342	0.01084551	0.43159634	-
	Hayfever	1.38797272	0.13717069	-0.204644	0.47898541	0.43154504	-
	Allergy	1.58894948	0.09656553	-0.1966512	0.38978222	0.51860972	-
	Pets	1.30499369	-0.1178061	-0.3943457	0.15873346	0.40373923	-
	Pollutants	1.3990406	-0.1443653	-0.5049772	0.21624666	0.43265591	-
	Alcohol	1.87415373	0.04500579	-0.2034652	0.29347676	0.7225771	-

Additional Table 4. Results of (generalized) linear mixed models used to calculate the effects of respiratory site and smoking status on alpha diversity, inter-individual variability, community recovery, and between-group distances.

Results of generalized linear mixed models comparing respiratory sites per smoke status

	smoke	contrast	ratio	SE	z.ratio	p.value	compare
Observed	NS	nose / oroph	0.132411	0.02729	-9.81002	3.06E-22	site
	NS	nose / BAL	0.144706	0.029916	-9.35016	1.31E-20	site
	NS	oroph / BAL	1.092855	0.158436	0.612481	0.540219	site
	AS	nose / oroph	0.232236	0.030431	-11.1422	2.34E-28	site
	AS	nose / BAL	0.381489	0.051128	-7.19043	9.69E-13	site
	AS	oroph / BAL	1.642683	0.197779	4.122343	3.75E-05	site
	FS6w	nose / oroph	0.2325	0.030237	-11.2174	1.00E-28	site
	FS6w	nose / BAL	0.329301	0.043117	-8.48344	3.28E-17	site
	FS6w	oroph / BAL	1.416352	0.168416	2.927328	0.003419	site
	FS1y	nose / oroph	0.190898	0.041631	-7.59361	9.33E-14	site
	FS1y	nose / BAL	0.250064	0.05489	-6.31444	4.07E-10	site
	FS1y	oroph / BAL	1.309936	0.268056	1.31933	0.187059	site
Evenness	NS	nose / oroph	0.678493	0.191253	-1.37605	0.253207	site
	NS	nose / BAL	0.592617	0.168587	-1.83918	0.197667	site
	NS	oroph / BAL	0.873432	0.209948	-0.56298	0.573446	site
	AS	nose / oroph	1.213494	0.241312	0.973079	0.495771	site
	AS	nose / BAL	0.897775	0.182929	-0.52923	0.596644	site
	AS	oroph / BAL	0.739827	0.142683	-1.56247	0.354529	site
	FS6w	nose / oroph	0.480951	0.087963	-4.00225	9.41E-05	site
	FS6w	nose / BAL	0.313212	0.060081	-6.05182	4.30E-09	site
	FS6w	oroph / BAL	0.651235	0.125738	-2.22132	0.026329	site
	FS1y	nose / oroph	1.110204	0.357932	0.324265	0.745738	site
	FS1y	nose / BAL	0.738498	0.249236	-0.89821	0.55361	site
	FS1y	oroph / BAL	0.665191	0.223361	-1.21411	0.55361	site
Individuality	NS	nose / oroph	1.452025	0.244136	2.21821	0.039811	site
	NS	nose / BAL	1.043154	0.177122	0.248825	0.803496	site
	NS	oroph / BAL	0.718414	0.095583	-2.48567	0.038793	site
	AS	nose / oroph	2.716484	0.355211	7.642451	6.39E-14	site
	AS	nose / BAL	1.658096	0.22402	3.74274	0.000182	site
	AS	oroph / BAL	0.610383	0.070319	-4.28515	2.74E-05	site
	FS6w	nose / oroph	5.001911	0.729697	11.03496	7.77E-28	site
	FS6w	nose / BAL	4.331949	0.634953	10.00187	2.24E-23	site
	FS6w	oroph / BAL	0.866059	0.095128	-1.3092	0.190465	site
	FS1y	nose / oroph	2.023949	0.455957	3.129647	0.002625	site
	FS1y	nose / BAL	2.260508	0.504789	3.652314	0.00078	site
	FS1y	oroph / BAL	1.11688	0.224287	0.550451	0.58201	site

Results of linear mixed models comparing between-group distances across smoke status

	effect	term	estimate	std.error	statistic	df	p.value
oroph vs BAL	fixed	(Intercept)	0.076549	0.007828	9.778878	31.85179	4.10E-11
	fixed	TimeAS	-0.01388	0.010106	-1.37376	31.85179	0.179107
	fixed	TimeFS6w	-0.01897	0.010106	-1.87737	31.85179	0.069652
	fixed	TimeFS1y	-0.02177	0.012428	-1.7516	40.89318	0.087341

Results of generalized linear mixed models comparing smoke status per site

	site	contrast	ratio	SE	z.ratio	p.value	compare
Observed	nose	NS / AS	0.56779	0.118152	-2.71998	0.019586	smokestatus
	nose	NS / FS6w	0.535055	0.110873	-3.01801	0.015266	smokestatus
	nose	NS / FS1y	0.622143	0.153155	-1.92785	0.107748	smokestatus
	nose	AS / FS6w	0.942347	0.133646	-0.41871	0.67543	smokestatus
	nose	AS / FS1y	1.095728	0.212103	0.47227	0.67543	smokestatus
	nose	FS6w / FS1y	1.162765	0.224445	0.781242	0.651991	smokestatus
	oroph	NS / AS	0.995847	0.135338	-0.03062	0.975573	smokestatus
	oroph	NS / FS6w	0.939501	0.127465	-0.45998	0.940299	smokestatus
	oroph	NS / FS1y	0.896949	0.162716	-0.5995	0.940299	smokestatus
	oroph	AS / FS6w	0.943418	0.111346	-0.4935	0.940299	smokestatus
	oroph	AS / FS1y	0.900689	0.152687	-0.617	0.940299	smokestatus
	oroph	FS6w / FS1y	0.954708	0.161113	-0.27465	0.940299	smokestatus
	BAL	NS / AS	1.496869	0.206449	2.924692	0.020688	smokestatus
	BAL	NS / FS6w	1.217603	0.1666	1.43893	0.225256	smokestatus
	BAL	NS / FS1y	1.075116	0.197046	0.395181	0.692709	smokestatus
	BAL	AS / FS6w	0.813433	0.098325	-1.70829	0.175167	smokestatus
	BAL	AS / FS1y	0.718243	0.124754	-1.90535	0.170203	smokestatus
	BAL	FS6w / FS1y	0.882977	0.151763	-0.7241	0.562806	smokestatus
Evenness	nose	NS / AS	0.670247	0.188206	-1.42488	0.231287	smokestatus
	nose	NS / FS6w	1.838744	0.497669	2.250387	0.048849	smokestatus
	nose	NS / FS1y	0.804261	0.26978	-0.6494	0.516083	smokestatus
	nose	AS / FS6w	2.743381	0.532926	5.195084	1.23E-06	smokestatus
	nose	AS / FS1y	1.199946	0.333244	0.656342	0.516083	smokestatus
	nose	FS6w / FS1y	0.437397	0.116667	-3.10019	0.005802	smokestatus
	oroph	NS / AS	1.198747	0.271934	0.79911	0.848454	smokestatus
	oroph	NS / FS6w	1.303398	0.294118	1.174247	0.848454	smokestatus
	oroph	NS / FS1y	1.315995	0.391585	0.922822	0.848454	smokestatus
	oroph	AS / FS6w	1.0873	0.202317	0.449812	0.874172	smokestatus
	oroph	AS / FS1y	1.097809	0.295096	0.347153	0.874172	smokestatus
	oroph	FS6w / FS1y	1.009665	0.270354	0.035921	0.971345	smokestatus
	BAL	NS / AS	1.01538	0.238316	0.06503	0.99434	smokestatus
	BAL	NS / FS6w	0.971819	0.229319	-0.12114	0.99434	smokestatus
	BAL	NS / FS1y	1.00224	0.316067	0.007094	0.99434	smokestatus
	BAL	AS / FS6w	0.957099	0.191015	-0.21971	0.99434	smokestatus
	BAL	AS / FS1y	0.987059	0.285296	-0.04507	0.99434	smokestatus
	BAL	FS6w / FS1y	1.031303	0.299326	0.106197	0.99434	smokestatus
Individuality	nose	NS / AS	0.388779	0.069892	-5.25518	4.44E-07	smokestatus
	nose	NS / FS6w	0.231667	0.044256	-7.65559	1.15E-13	smokestatus
	nose	NS / FS1y	0.34026	0.07817	-4.69254	5.40E-06	smokestatus
	nose	AS / FS6w	0.595884	0.096191	-3.20711	0.002011	smokestatus
	nose	AS / FS1y	0.875202	0.178956	-0.65192	0.514454	smokestatus
	nose	FS6w / FS1y	1.468747	0.316457	1.78413	0.089283	smokestatus
	oroph	NS / AS	0.727338	0.092991	-2.49012	0.019155	smokestatus
	oroph	NS / FS6w	0.798043	0.101606	-1.77188	0.091698	smokestatus
	oroph	NS / FS1y	0.474282	0.084451	-4.1893	0.000168	smokestatus
	oroph	AS / FS6w	1.097212	0.120188	0.846925	0.397037	smokestatus
	oroph	AS / FS1y	0.65208	0.108276	-2.5751	0.019155	smokestatus
	oroph	FS6w / FS1y	0.594306	0.098413	-3.14239	0.005027	smokestatus
	BAL	NS / AS	0.617965	0.08302	-3.58278	0.00102	smokestatus
	BAL	NS / FS6w	0.962054	0.125287	-0.29706	0.766424	smokestatus
	BAL	NS / FS1y	0.737342	0.130622	-1.72001	0.155518	smokestatus
	BAL	AS / FS6w	1.556808	0.1798	3.832593	0.000761	smokestatus
	BAL	AS / FS1y	1.193176	0.198926	1.059377	0.347314	smokestatus
	BAL	FS6w / FS1y	0.766425	0.125291	-1.62727	0.155518	smokestatus
Recovery	nose	AS / FS6w	1.180213	0.236208	0.827894	0.784885	smokestatus
	nose	AS / FS1y	1.076263	0.288911	0.273785	0.784885	smokestatus
	nose	FS6w / FS1y	0.911923	0.248983	-0.33769	0.784885	smokestatus
	oroph	AS / FS6w	0.914507	0.128952	-0.6338	0.527837	smokestatus
	oroph	AS / FS1y	1.223627	0.25525	0.967489	0.503882	smokestatus
	oroph	FS6w / FS1y	1.338018	0.277881	1.402099	0.493092	smokestatus
	BAL	AS / FS6w	0.789238	0.11853	-1.576	0.355722	smokestatus
	BAL	AS / FS1y	0.878733	0.190644	-0.59586	0.617195	smokestatus
	BAL	FS6w / FS1y	1.113394	0.238429	0.50159	0.617195	smokestatus
